# Cross-cultural variation in experiences of acceptance, camouflaging and mental health difficulties in autism: A registered report

**DOI:** 10.1371/journal.pone.0299824

**Published:** 2024-03-20

**Authors:** Connor Tom Keating, Lydia Hickman, Philippine Geelhand, Toru Takahashi, Joan Leung, Ruth Monk, Bianca Schuster, Alicia Rybicki, Teresa Marie Girolamo, Elise Clin, Fanny Papastamou, Marie Belenger, Inge-Marie Eigsti, Jennifer Louise Cook, Hirotaka Kosaka, Rieko Osu, Yuko Okamoto, Sophie Sowden-Carvalho

**Affiliations:** 1 Centre for Developmental Science, School of Psychology, University of Birmingham, Birmingham, United Kingdom; 2 ACTE (Autism in Context: Theory and Experiment) at LaDisco (Center for Linguistics Research) and ULB Neuroscience Institute, Université libre de Bruxelles, Brussels, Belgium; 3 Faculty of Human Sciences, Waseda University, Tokorozawa, Japan; 4 School of Psychology, University of Auckland, Auckland, New Zealand; 5 Autistic Member of the Autism New Zealand Community Advisory Group, New Zealand/School of Medical Sciences, University of Auckland, Auckland, New Zealand; 6 Department of Cognition, Emotion and Methods in Psychology, University of Vienna, Vienna, Austria; 7 School of Speech, Language, and Hearing Sciences, San Diego State University, San Diego, CA, United States of America; 8 Department of Psychological Sciences, University of Connecticut, Storrs, CT, United States of America; 9 Department of Neuropsychiatry, University of Fukui, Fukui, Japan; 10 Waseda Institute for Advanced Study, Waseda University, Tokyo, Japan; Public Library of Science, UNITED STATES

## Abstract

Recent findings suggest that stigma and camouflaging contribute to mental health difficulties for autistic individuals, however, this evidence is largely based on UK samples. While studies have shown cross-cultural differences in levels of autism-related stigma, it is unclear whether camouflaging and mental health difficulties vary across cultures. Hence, the current study had two aims: (1) to determine whether significant relationships between autism acceptance, camouflaging, and mental health difficulties replicate in a cross-cultural sample of autistic adults, and (2) to compare these variables across cultures. To fulfil these aims, 306 autistic adults from eight countries (Australia, Belgium, Canada, Japan, New Zealand, South Africa, the United Kingdom, and the United States) completed a series of online questionnaires. We found that external acceptance and personal acceptance were associated with lower levels of depression but not camouflaging or stress. Higher camouflaging was associated with elevated levels of depression, anxiety, and stress. Significant differences were found across countries in external acceptance, personal acceptance, depression, anxiety, and stress, even after controlling for relevant covariates. Levels of camouflaging also differed across countries however this effect became non-significant after controlling for the covariates. These findings have significant implications, identifying priority regions for anti-stigma interventions, and highlighting countries where greater support for mental health difficulties is needed.

## Introduction

Autism spectrum disorder (hereafter ‘autism’) is a neurodevelopmental condition that is characterised by difficulties in social communication and restricted and repetitive interests [1]. Throughout life, autistic people (‘identity-first’ terminology is used throughout in line with the preferences of the autistic community [2, 3] and guidance for avoiding ableist language [4]) experience a higher risk of psychiatric and mental health disorders [5] and elevated risks of premature mortality by nearly two decades relative to their non-autistic peers [6]. In a population-derived sample of young autistic people aged 10 to 14, 71% met criteria for at least one mental health disorder, with 41% having additional psychiatric diagnoses [7]. Similarly high rates of mental health problems have been found in other studies of autistic adolescents [8] and autistic adults [9–12]. Two of the most common psychiatric problems for autistic adults are depression and anxiety [13–16], with prevalence rates for depressive and anxiety disorders reaching as high as 47.1% and 54.0%, respectively [17]. It is also possible that prevalence rates of co-occurring psychiatric and mental health difficulties are underestimated, with many autistic individuals being subject to missed, delayed or inaccurate diagnoses [18] and healthcare needs which remain unmet in health systems modelled on the needs of neurotypical individuals [19].

For autistic individuals, mental health difficulties increase the likelihood of poorer long-term outcomes [20] including lower social and adaptive functioning [11], educational and employment difficulties [21, 22], poorer quality of life [23–25], and suicidality [26]. To support autistic people in achieving positive long-term outcomes, it is important to further understand the presentation of mental ill-health in autism and to determine *why* autistic individuals are at higher risk of mental health difficulties. This is crucial since the mental health of autistic individuals has been identified as a key priority for research across multiple stakeholder groups [27, 28].

There are many factors that could contribute to elevated mental health difficulties in autism. For example, since the prevalence of alexithymia (a subclinical condition characterised by difficulties identifying, expressing and differentiating emotions [29]), a transdiagnostic risk factor for numerous psychiatric conditions (e.g., depression, [30], and anxiety disorders, [31]) is higher in the autistic [49.93%] than the non-autistic population (4.89%; [32]), alexithymia may lead to increased risk of mental health difficulties in autism. Alternatively, Botha and Frost [33] advocate the extension of the minority stress model to explain the substantial health disparities between autistic and non-autistic individuals. The minority stress model posits that high stress burdens can negatively impact the mental and physical health of marginalised groups [34]. Such stress burden and resultant poorer health is frequently observed in minority groups including sexual, gender identity and ethnic minorities [35, 36], and minority stressors have recently been described to increase depressive symptoms in those with physical disabilities [37]. Examples of minority stressors include everyday discrimination, internalised stigma, and concealment. In line with this, the current study focuses on the impact of autism acceptance and camouflaging (minority stressors) on the mental health of autistic individuals (a marginalised group).

### Autism acceptance, camouflaging and mental health

Autism acceptance, as discussed here, can be defined as “an individual feeling accepted or appreciated as an autistic person, with autism positively recognised and accepted by others and the self as an integral part of that individual” [38]. The majority of autistic individuals in the UK feel that society does not accept [43%] or only sometimes accepts [48%] them as an autistic person (in total 91%; [38]). In spite of this, until recently, research had not directly examined autistic people’s experiences of autism acceptance and how this relates to mental health difficulties. The first study to do so confirmed a relationship between acceptance and mental health, finding that autism acceptance from external sources (i.e., from family, friends, and society) was associated with depression and stress [38] and personal acceptance (i.e., acceptance of oneself as an autistic person) was associated with depression. In addition, individuals who spontaneously reported camouflaging their autistic traits also reported higher symptoms of depression and fewer experiences of acceptance in the past week [38].

Camouflaging can be defined as “the use of strategies by autistic people to minimise the visibility of their autism in social situations” [39]. These strategies can involve compensation, whereby autistic individuals develop behaviours to help them manage social situations (e.g., learning how to use eye contact or developing scripts to help them navigate social interactions; [39, 40]), and masking, whereby autistic individuals aim to hide their autistic traits (e.g., deliberately suppressing stimming behaviours; [39]). Whilst the vast majority of autistic individuals report camouflaging to some degree [39], there are indications that camouflaging may be more common among women [41–49] and those higher in autistic traits [41, 50–52]. There is inconsistent evidence regarding whether camouflaging is associated with broader cognitive ability [41], with some studies finding that a higher level of education is associated with greater camouflaging [50], and others suggesting there is no association between camouflaging and Intelligence Quotient (IQ) [39, 43, 53] or executive functioning [49].

However, a burgeoning literature suggests that camouflaging may be a risk factor for depression and anxiety in autism [26, 38, 54–58], irrespective of gender [59]. Interestingly, Cage and colleagues [38] suggest that camouflaging may mediate the relationship between social stressors (e.g., lack of autism acceptance, bullying) and anxiety and depression. That is, those who experience more social stressors (such as a lack of autism acceptance) are more likely to camouflage their autistic traits, which in turn results in higher levels of anxiety and depression. However, a recent study found that whilst autism-related stigma was associated with lower wellbeing, camouflaging did not mediate this relationship (despite camouflaging being positively associated with autism-related stigma; [60]). Future research should verify whether camouflaging mediates the relationship between autism acceptance/stigma and anxiety and depression more specifically.

It is important to note that the terms we use to refer to camouflaging vary across the globe [61]. In Japan, the term ‘over-adaptation’ is used to describe excessive efforts made by people (i.e., society more broadly) to meet external demands and expectations, even if this means forcibly suppressing their internal needs [62]. In recent years, this term has been applied to autism to describe the substantial efforts made by autistic people to fit into an environment as a result of differences in interpersonal and social behaviour [63–65]. As such, this concept is highly similar to the construct of camouflaging (see [62] for a discussion around the similarities and differences between the constructs of camouflaging and over-adaptation in autism). Importantly, there is anecdotal evidence that over-adaptation is associated with poorer mental health in autistic individuals in Japan [66]. However, to date no empirical studies have tested this relationship [62], and therefore further study of over-adaptation and camouflaging in Japan is necessary.

### Cross-cultural perspectives on autism acceptance, camouflaging and mental health

Culture can be defined as “a set of basic assumptions and values, orientations to life, beliefs, policies, procedures and behavioural conventions that are shared by a group of people” [67]. Unsurprisingly, across different cultures there can be large variation in these beliefs, attitudes and behaviours. In the case of autism, we see cross-cultural variation in attitudes towards autistic people: previous research has found that *non-autistic* college students in the US reported lower levels of autism-related stigma than their Lebanese [68, 69] and Japanese [70] counterparts. Whilst these studies demonstrate that there may be variation in levels of autism stigmatisation among *non-autistic* individuals across cultures, studies have not yet compared *autistic* individuals’ experiences of autism acceptance across different cultures. Given that levels of stigma reported by non-autistic individuals vary cross-culturally, it is likely that acceptance from the perspective of autistic individuals themselves also vary across cultures. Such cross-cultural differences may exist in both external acceptance (as experiencing stigma may result in autistic individuals feeling less accepted by others) and personal acceptance (as stigma can become internalised resulting in decreased personal acceptance or ‘self-stigma’; [55, 71, 72]).

As Lai and colleagues [73] highlight, it is paramount to determine levels of camouflaging in the autistic population. Currently, it is unknown how many undiagnosed autistic adults exist [74]. However, it is thought that there are more undiagnosed autistic individuals in some areas of the world than others [75]. Investigating the rates of camouflaging across cultures will help us work towards determining the true prevalence of autism, and its sex/gender ratio, across the globe. To our knowledge, studies have not yet explored camouflaging in autistic populations outside of the UK (e.g., [59]) and USA (e.g., [76]), though there are anecdotal reports of over-adaptation in Japan. Rather than assuming levels of camouflaging in the UK and USA reflect those globally, researchers should collaborate internationally to compare camouflaging behaviours cross-culturally. Cross-cultural perspectives are likely to have great utility for understanding the mechanisms underlying camouflaging, as the social environments autistic individuals aim to adapt to vary across cultures. For example, by determining whether rates of camouflaging are higher within certain cultures, we can work towards identifying socio-cultural factors that may contribute to camouflaging. This is important as there is a consensus among researchers that ‘there is much to be learnt about [the] mechanisms’ involved in autistic camouflaging [73].

Whilst no research has compared camouflaging across cultures, it seems likely that rates of camouflaging will vary between cultures for a number of reasons. Firstly, in collectivist cultures (which prioritise community interdependence and shared group norms and values; [77]), there may be more pressure relative to individualistic cultures (which prioritise independence and individualism; [77]) to camouflage one’s autistic traits in order to minimise deviation from shared group norms. Thus, those in collectivist cultures may alter their behaviour or hide their autistic traits in order to avoid shame and fit in [70]. By contrast, in regions where there are more discussions about neurodiversity–both as a movement and as an ideology (e.g., the United States, Canada, and the United Kingdom; see [78])–individuals may feel less pressured to camouflage their autistic traits. Finally, since autism-related stigma varies across cultures [68–70] and reduced autism acceptance is associated with increased camouflaging [38, 60], levels of camouflaging may also differ cross-culturally.

Moreover, to the best of our knowledge, studies have not yet compared the level of mental health difficulties in cross-cultural samples of autistic individuals. Like camouflaging, there are reasons to believe there is cross-cultural variation in the levels of these difficulties. Firstly, since autism acceptance differs across cultures [68–70], the level of mental health difficulties may also vary because a lack of autism acceptance or stigma is associated with poorer mental health [33, 38]. Secondly, since beliefs about mental health vary across cultures [79], and these beliefs can affect willingness to seek and adhere to treatment [80], there may be cross-cultural differences in levels of mental health difficulties. Finally, given that accessibility of mental health services also differs across cultures [81], individuals from certain regions may be unable to get the help they need, thus leading to poorer mental health. As such, research is necessary to explore whether the levels of mental health difficulties experienced by autistic individuals vary across cultures.

### The current study

In the current study, we aimed to replicate the regression analyses from previous research, which found relationships between experiences of acceptance, camouflaging and mental health, in a diverse sample of autistic adults. In addition, we aimed to compare autistic individuals’ levels of autism acceptance, camouflaging and mental health across multiple countries: Australia, Belgium, Canada, Japan, New Zealand, South Africa, the United Kingdom, and the United States. Importantly, exploring these factors across cultures has the potential to a) elucidate the potential routes to greater autism acceptance, reduced need for camouflaging behaviours, and improved mental health outcomes, b) identify ‘priority regions’ for anti-stigma interventions (like those in Ranson and Byrne, [82] and Gillespie-Lynch et al., [83]), and c) highlight areas where greater support for mental health difficulties is needed. In addition, since acceptance is likely to vary across cultures, we employed an experimental design which naturally maximises variation in autism acceptance, thus giving us the ability to more sensitively assess the impact of autism acceptance on camouflaging and mental health respectively.

To fulfil our aims, participants completed a number of questionnaires including Autism Acceptance Questions [38], the Camouflaging Autistic Traits Questionnaire [52], and the 21-item Depression, Anxiety and Stress Scale (DASS-21; [84]). Participants also provided demographic information.

### Hypotheses

#### Replicating the regression analyses from previous research

**Fig 1:**
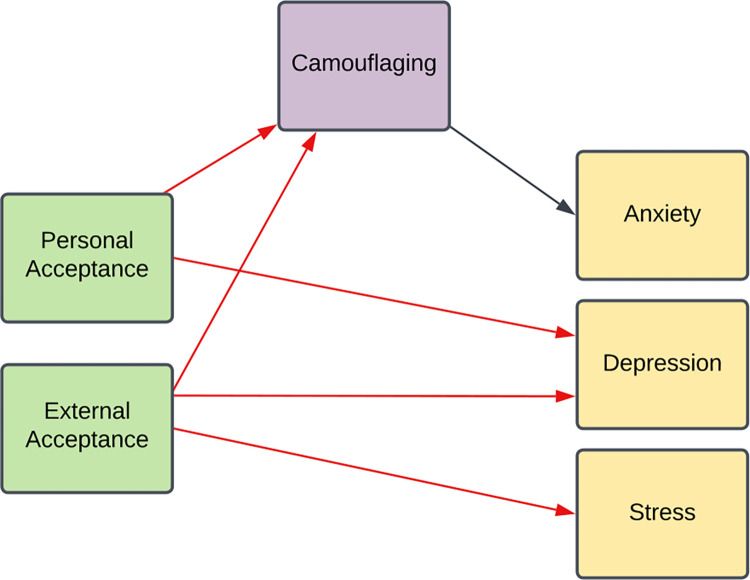
A visual representation of the relationships that were tested in the current study. Black arrows indicate positive relationships. Red arrows indicate negative relationships.

In line with Cage and colleagues [38], we predicted that external and personal acceptance would be associated with depression scores after controlling for age, gender, age of diagnosis, anxiety scores and stress scores. In addition, we predicted that there would be an association between external acceptance and stress scores after controlling for age, gender, age of diagnosis, anxiety scores and depression scores. As was the case in Perry et al., [60], we hypothesized that autism acceptance would be associated with camouflaging after controlling for age, gender, age of diagnosis, and autistic traits. In line with Hull et al., [59] we predicted that there would be an association between camouflaging and anxiety scores after controlling for age and autistic traits (see Fig 1 for a visual representations of these hypotheses).

#### Comparing levels of acceptance, camouflaging and mental health across cultures

We predicted that there would be differences in the level of autism acceptance across countries. In addition, we aimed to explore whether there were any differences in levels of camouflaging and mental health difficulties across countries.

## Materials and methods

The registered report protocol for this study [85] was published online prior to data collection. There were no deviations to the registered procedures or primary statistical analyses.

### Participants

309 participants were recruited for the current study. These participants were recruited between January 2022 and October 2022. Three participants did not have a formal diagnosis of autism from an independent clinician and therefore were excluded. As such, the final sample comprised 306 autistic adults from eight countries across the globe: Australia (N = 40), Belgium (N = 38), Canada (N = 40), Japan (N = 35), New Zealand (N = 31), South Africa (N = 40), the UK (N = 42), and the USA (N = 40). These countries vary in cultural orientation, both in terms of individualism-collectivism (individualism scores: Japan = 46; South Africa = 65; Belgium = 75; New Zealand = 79; Canada = 80; UK = 89; Australia = 90; USA = 91 [86, 87]), and in verticality-horizontality (verticality scores: Belgium = 65; Japan = 54; South Africa = 49; USA = 40; Canada = 39; Australia = 38; UK = 35; New Zealand = 22 [86, 87]). While individualistic cultures prioritise independence and distinctiveness [77], collectivist cultures prioritise community interdependence and shared group norms [77]. Relatedly, while horizontal cultures value equality amongst its members, vertical cultures value hierarchy [88–90]. Here, we included countries which vary across these cultural dimensions, thus allowing us to test the putative relationships between acceptance, camouflaging and mental health in a more diverse sample (than employed previously).

Participants’ gender, age, level of autistic traits, and level of alexithymic traits are detailed in Table 1. Information regarding participants’ levels of education, income, current verbal level, childhood verbal level, sexuality, autism diagnosis, age of diagnosis, race, and ethnicity are detailed in S1 File.

**Table 1:** Participants’ genders, ages, and levels of autistic traits and alexithymic traits. Means are followed by standard deviations in parentheses.

Country	Gender	Age	AQ	TAS
Australia (N = 40)	20 Female, 13 Male, 6 Non-binary/Third gender, 1 Choose not to disclose	28.35(7.40)	35.15(7.01)	62.83(13.12)
Belgium (N = 38)	17 Female, 16 Male, 2 Non-binary/Third gender, 2 Other, 1 Choose not to disclose	38.87(9.38)	37.16(8.14)	64.74(14.37)
Canada (N = 40)	20 Female, 14 Male, 4 Non-binary/Third gender, 1 Other, 1 Choose not to disclose	28.48(9.26)	34.08(7.47)	63.45(11.27)
Japan (N = 35)	11 Female, 20 Male, 2 Non-binary/Third gender, 1 Other, 1 Choose not to disclose	30.63(7.46)	29.20(6.47)	52.34(9.81)
New Zealand (N = 31)	11 Female, 17 Male, 1 Non-binary/Third Gender, 2 Other	30.84(8.38)	36.52(6.73)	61.55(12.92)
South Africa (N = 40)	20 Female, 18 Male, 1 Non-binary/Third gender, 1 Choose not to disclose	26.18(6.17)	29.18(8.40)	63.80(15.32)
UK (N = 42)	15 Female, 18 Male, 5 Non-binary/Third gender, 3 Other, 1 Choose not to disclose	31.71(10.77)	35.40(8.54)	62.00(13.75)
USA (N = 40)	19 Female, 15 Male, 5 Non-binary/Third gender, 1 Other	31.05(8.36)	35.42(6.82)	58.40(11.30)

### Sample size rationale

In our regression analyses, we planned to include data for all participants who completed the study in one group (regardless of country of residence) to replicate previous findings [38, 59, 60] in a large and cross-cultural sample of autistic adults. The chosen sample size for these analyses was based on sample size calculations conducted using G*Power [91], which focused on replicating the regression analyses of interest from Cage et al., [38], Perry et al., [60] and Hull et al., [59].

For the following sample size calculations, we used a Bonferroni-adjusted alpha level of 0.0125 to account for multiple testing (4 regression analyses), and a power level of 90% (thereby giving us higher power than is standard in the field [92, 93]). To replicate the finding from Cage et al., [38] that external acceptance and personal acceptance account for 17.5% variance explained in depression scores after controlling for age, gender, age of diagnosis, anxiety and stress (f^2^ = 0.37), 51 participants are required. To replicate the finding that external acceptance and personal acceptance account for 3% variance explained in stress scores after controlling for age, gender, age of diagnosis, anxiety and stress, 246 participants are required. To replicate the significant association between stigma and camouflaging (f^2^ = 0.069) after controlling for age, gender, age of diagnosis, and autistic traits (as found in Perry et al., [60]), 211 participants are required. Finally, to replicate the finding that anxiety accounts for 4% variance explained in camouflaging scores after controlling for age and autistic traits (as in Hull et al. [59]), 286 participants are required. Therefore, we aimed to ensure that our final sample comprised at least 286 participants.

To facilitate the analyses comparing participants’ experiences of autism acceptance, camouflaging, and levels of depression, anxiety and stress across countries, we ensured that each group had at least 30 participants (and therefore we had sufficient data to draw comparisons between these multiple groups). We selected this cut-off as Sekaran [94] highlights that “where samples are to be broken into sub-samples…a minimum sample size of 30 for each category is necessary”. Importantly, Sekaran [94] also highlights that “in multivariate research…the sample size should be several times (preferably 10 times or more) as large as the number of variables in this study”. By recruiting 306 participants to our study, our sample was: (1) 51 times larger than the number of variables in our multivariate analysis (6 variables: external acceptance, personal acceptance, camouflaging, depression, anxiety and stress), (2) met the required sample size based on our power analyses (largest sample size generated = 286), and (3) facilitated comparisons between participants in 8 countries.

Participants were recruited via an existing international autism research database kept by the U21 Autism Research Network. The database was set up as part of a collaboration between seven autism research groups across 4 continents and 7 countries (Australia, Belgium, Hong Kong, Japan, New Zealand, UK, and USA). The aim of this initiative was to diversify samples recruited for autism research and to generate opportunities to answer questions relevant to the global autistic community. In the current study, we recruited participants from our sites in Australia, Belgium, Japan, New Zealand, the UK and the USA. In addition, we recruited participants via social media and Prolific, thus allowing us to recruit participants from Canada, and South Africa. All participants provided informed consent. This study was approved by the Science, Technology, Engineering and Mathematics (STEM) ethics committee at the University of Birmingham (ERN_16-0281AP9D).

### Materials and stimuli

#### Demographic information

Participants were asked a number of demographic questions to establish the nature of the sample. These included questions about a) age, b) sex assigned at birth, c) gender identity, d) sexual identity, e) ethnicities, f) country of birth, g) country of residence, h) years lived in country of residence, i) level of education, j) number of siblings, k) income, l) co-occurring diagnoses, m) how verbal they would consider themselves now, and n) how verbal they were as a child (as recommended by Botha, Hanlon and Williams [95]). Participants were also asked to confirm whether they have an autism diagnosis, which diagnosis they received (e.g., Autism vs. Autism Spectrum Disorder), who gave them their diagnosis (e.g., multidisciplinary team, doctor, clinical psychologist, etc.), and finally the age they received that diagnosis. This information, along with participants’ questionnaire scores, can be found at https://osf.io/q8tew/. All identifying information was stored separately in a password protected database prior to conducting any statistical analyses on the questionnaire data.

#### Autism acceptance

To measure perceptions of autism acceptance, we used the self-report autism acceptance questions outlined in Cage et al., [38]. First, to obtain a categorical response for acceptance, participants were asked whether they feel that society (specified as the general public, made up of people who do not personally know them) generally accepted them, with “yes”, “no”, “sometimes” and “prefer not to say” as response options. Second, participants were asked to respond to the statement “over the past week, I have felt accepted by society as an autistic person/ person with autism”, on a five-point Likert scale from “strongly agree” to “strongly disagree”. Next, to measure perceptions of autism acceptance from different sources, participants were also asked: “how accepted by society do you feel as an autistic person?”; “how accepted by your family and friends do you feel as an autistic person?”; and “how much have you personally accepted yourself as an autistic person?”. They reported their responses on a scale from zero, “not at all”, to ten, “completely”. The English version of the autism acceptance questions has acceptable internal consistency (Cronbach’s α = 0.64) and good validity [38]. These questions also had acceptance internal consistency here (McDonald’s ωt = 0.71). In the current study, we translated the autism acceptance questions into French and Japanese to allow us to collect data in Belgium and Japan (see information below).

#### Camouflaging of autistic traits

To measure the extent to which participants camouflage their autistic traits, we used the Camouflaging Autistic Traits Questionnaire (CAT-Q; [52]). The CAT-Q comprises 25 items (e.g., “In social situations, I feel like I’m pretending to be normal”), rated on a seven-point Likert scale (ranging from one, “Strongly Disagree” to seven, “Strongly Agree”). Total scores on the CAT-Q can range from 25–175, with higher scores reflecting greater camouflaging. This self-report questionnaire assesses three different domains relevant to camouflaging: compensation (strategies used to compensate for socio-communicative difficulties, e.g., “I have spent time learning social skills from television shows and films, and try to use these in my interactions”); masking (strategies used to present a non-autistic or less autistic persona to others, e.g., “I monitor my body language or facial expressions so that I appear interested by the person I am interacting with”), and assimilation (strategies used to fit in to uncomfortable social situations, e.g., “I have to force myself to interact with people when I am in social situations”). The English and Japanese versions of the CAT-Q have strong psychometric properties, including internal consistency (English Cronbach’s α = 0.94; Japanese Cronbach’s α = 0.87) and test-retest reliability (English Pearson’s r = 0.77; Japanese Pearson’s r = 0.82 [52, 96]). The CAT-Q also had strong internal consistency here (McDonald’s ωt = 0.93). In the current study, we translated the CAT-Q into French to allow us to collect data in Belgium (see information below).

#### Depression, anxiety and stress

To measure levels of depression, anxiety and stress, we used the Depression, Anxiety and Stress Scale (DASS-21; [84]). In this 21-item questionnaire, participants judge whether certain statements (e.g., “I found it hard to wind down”) could be applied to their life over the past week on a scale from zero to three (0 = “did not apply to me at all; 1 = “applied to me some of the time”; 2 = “applied to me a considerable degree”; 3 = “applied to me very much or most of the time”). The 21 items can be reduced to 7 items each for the depression, anxiety and stress subscales. Total scores for each subscale are calculated and multiplied by two (meaning that scores for each subscale range from zero to 42), with higher scores representing higher depression, anxiety and stress. The DASS-21 has been previously used with autistic participants (e.g., [38, 97]) and the English [38, 97], French [98], and Japanese [99] versions have good internal consistency and factor validity. This scale also had strong internal consistency here (McDonald’s ωt = 0.95).

#### Questionnaires to facilitate controlling for other participant characteristics

The following questionnaires were included to facilitate a) the exploration of the relationships found previously between autistic traits and camouflaging, and alexithymic traits and mental health (see S4 File), and b) our analyses in which we control for these participant characteristics (see Exploratory Analyses).

#### Autistic traits

The autistic traits of all participants were assessed via the Autism Quotient (AQ; [100]). This self-report questionnaire assesses five domains relevant to autistic characteristics (attention switching, attention to detail, communication, social skill and imagination). In this questionnaire, participants are instructed to respond to 50 items with one of four responses: ‘definitely agree’, ‘slightly agree’, ‘slightly disagree’ and ‘definitely disagree’. These responses are scored using a binary system, where endorsing an autistic trait (either mildly or strongly) is scored as 1, and not endorsing an autistic trait (either mildly or strongly) is scored as 0. As such, scores on the AQ range from 0 to 50, with higher scores representing higher levels of autistic traits. The English [101], French [102], and Japanese [103] versions of the AQ have strong psychometric properties. The AQ also had good internal consistency here (McDonald’s ωt = 0.88).

#### Alexithymic traits

The alexithymic traits of participants was measured via the 20-item Toronto Alexithymia Scale [104]. This self-report questionnaire comprises 20 items rated on a five-point Likert scale (from 1, strongly disagree, to 5, strongly agree). Total scores on the TAS-20 range from 20 to 100, with higher scores representing higher levels of alexithymic traits. The TAS-20 is the most popular tool for assessing alexithymia and the English [104, 105], French [106], and Japanese [107] versions have good psychometric properties. The TAS-20 also had good internal consistency here (McDonald’s ωt = 0.82).

### Translation of relevant questionnaires

Many of the questionnaires used in this study (e.g., the Depression, Anxiety and Stress Scale, the Autism Quotient, the Toronto Alexithymia Scale) had already been translated into the target languages (English, French, Japanese). However, the CAT-Q was yet to be translated into French and the Autism Acceptance Questions were yet to be translated into French and Japanese. We followed the recommendations outlined in Gorecki et al., [108] for conducting translations for cross-cultural research. As such, we used the forward-backward method of translation that has been used by other researchers in this field (e.g., [70]).

To check the psychometric properties of the translated versions of the CAT-Q and autism acceptance questions, we conducted item and factor analyses. Our item analyses demonstrated good internal consistency for the French and Japanese versions of the autism acceptance questions and the CAT-Q, across all subscales (see S2 File). Our Principal Component Analysis revealed a different factor structure for the French version of the CAT-Q relative to the English version (see S2 File). These results are in line with several other studies that have documented factor structures that differ from the original English version of the CAT-Q (e.g., Japanese [96]; Swedish [109]). Nevertheless, these previous studies have concluded that the translated versions of the CAT-Q are appropriate for use due to their high reliability. Thus, although the total scores on these scales appear to be reliable indicators of acceptance and camouflaging, caution should be advised when interpreting the scores on the individual subscales of the CAT-Q (due to there being differences in factor structure). As a result, in the current study we did not conduct any analyses involving the individual subscales of the CAT-Q.

### Community involvement

Following participatory research guidelines [110, 111], the proposal for this study was reviewed by members of the autism community (within the Birmingham Psychology Autism Research Team Consultancy Committee) to ensure that it was in line with their research priorities. The feedback from the community was positive and their advice was actioned.

## Results

### Experiences of acceptance

**Fig 2:**
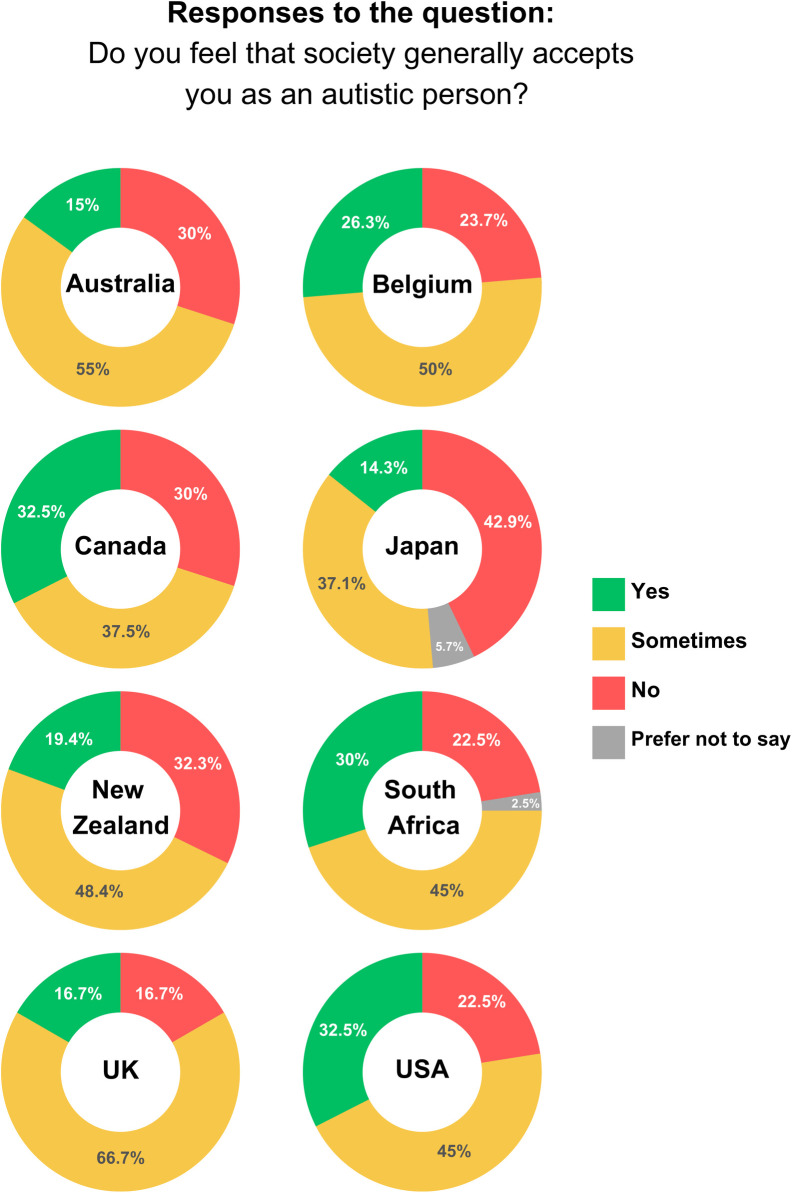
Responses to the question “do you feel that society generally accepts you as an autistic person” across country groups.

**Fig 3:**
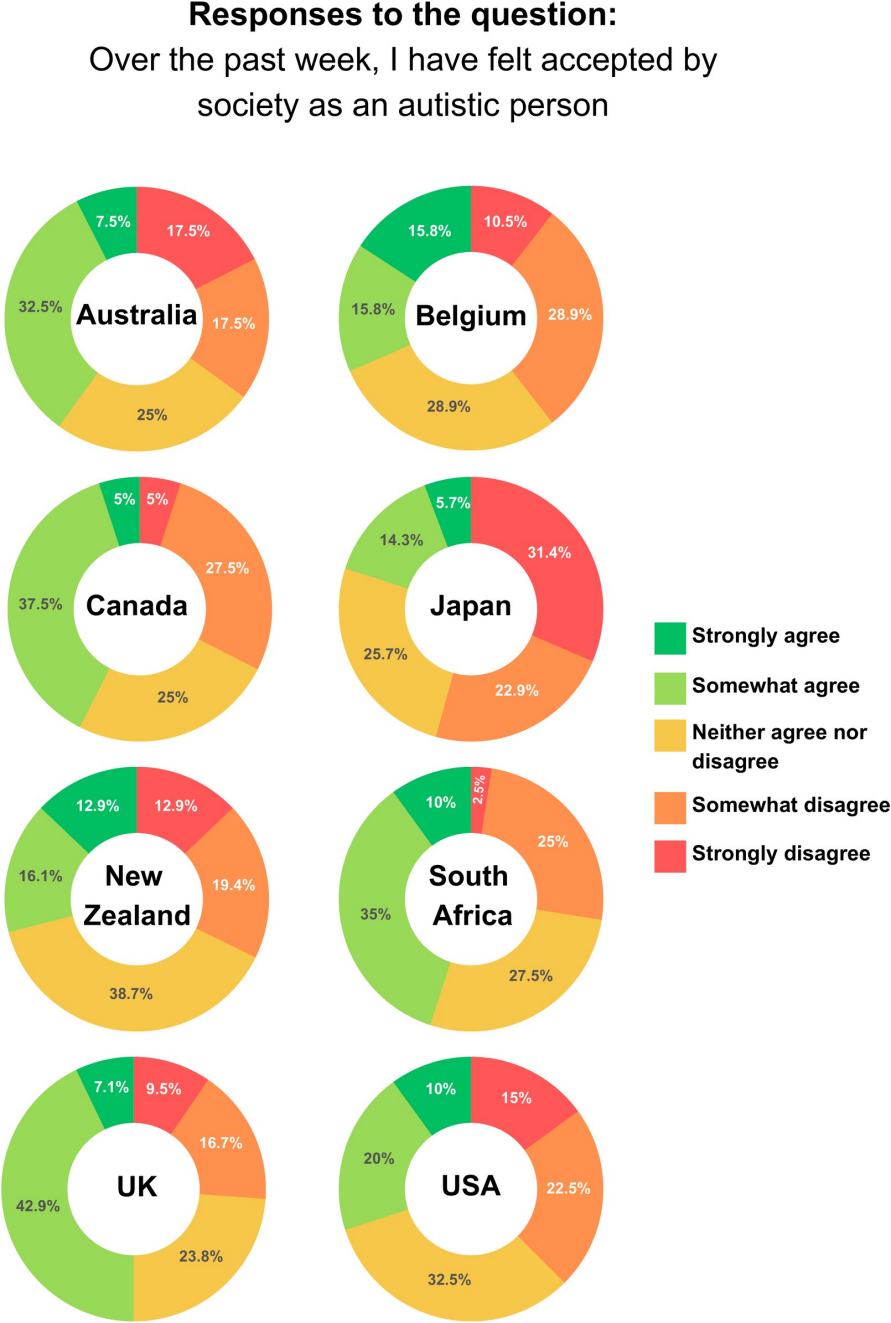
Responses to the question “over the past week I have felt accepted by society as an autistic person” across country groups.

Across country groups, 23.5% of participants felt that society accepted them as an autistic person, 48.4% only sometimes felt accepted, and 27.1% did not feel accepted (1.0% prefer not to say; see Fig 2 for variation across countries). Participants were also asked to rate the statement “over the past week, I have felt accepted by society as an autistic person”– 36.7% strongly agreed or agreed, 28.1% neither agreed nor disagreed, and 35.2% disagreed or strongly disagreed (see Fig 3 for variation across countries).

As stated in our registered report protocol [85], here we first include the data for all participants who completed the study in one group (collapsing across countries) to explore relationships in a large and cross-cultural sample of autistic adults. As registered, for these four regression analyses, we used a p < .0125 threshold to establish whether to reject the null hypothesis (corrected for multiple testing). Following this, we then separate participants into groups based on their country of residence and compare experiences of autism acceptance, camouflaging, and levels of depression, anxiety and stress across these groups. There was no missing data on our primary outcome measures for any participants.

### Registered analyses: Aiming to replicate previous results in a more diverse sample of autistic adults

#### Do personal acceptance and external acceptance significantly relate to depression and stress?

Following the procedures from Cage et al. [38], we conducted two hierarchical regression analyses with depression and stress as the outcome variables respectively. In step one, age, age of diagnosis, gender, and the other DASS subscales were entered; in step two, external acceptance (i.e., the average of the scores for acceptance from family and friends, and acceptance from society) and personal acceptance were added.

First, depression was considered as the outcome variable; the variables in step one accounted for 50.3% of the variance in depression scores. Depression was significantly related to anxiety [t(295) = 4.29, p < .0001] and stress [t(295) = 7.65, p < .0001], but no other variables [all p > .0125]. In the second step, adding personal and external acceptance significantly improved the model [F(2,293) = 11.86, p < .0001, R^2^ change = 3.41%]. Those who experienced greater personal acceptance [t(293) = -3.13, p = .0019; see S3 File for exploratory analyses comparing the strength of this association across our country groups], lower anxiety [t(293) = 4.00, p < .0001] and lower stress [t(293) = 7.40, p < .0001] typically had lower depression scores. External acceptance was not significantly related to depression at our revised p < .0125 threshold [t(293) = -2.03, p = .0430], nor were any of the other variables.

Notably, we identified that external acceptance and personal acceptance were moderately correlated [r = .371, p < .0001] and loaded on to the same factor in our aggregated sample [factor loadings: acceptance from family and friends = 0.84; acceptance from society = 0.84; personal acceptance = 0.68]. Therefore, it was plausible that personal acceptance explained the same portion of variance as external acceptance in depression scores, leading to a non-significant association between external acceptance and depression at our revised p < .0125 threshold. As such, we conducted one further post-hoc multiple regression analysis–this time excluding personal acceptance–to verify whether this was the case. This analysis revealed that, when personal acceptance was not included in the model, there was a significant association between external acceptance and depression [t(294) = -3.68, p = .0003]: those that experienced higher levels of external acceptance typically experienced lower levels of depression. Hence, both external and personal acceptance are associated with depression scores but may account for overlapping variance; when tested together, external acceptance is no longer significantly related to depression as personal acceptance has greater explanatory power.

In the second hierarchical regression, stress was included as the outcome variable. The variables in step one accounted for 66.1% of the variance in stress scores. Stress was significantly related to both depression [t(295) = 7.65, p < .0001] and anxiety [t(295) = 12.17, p < .0001], but none of the other predictors [all p > .0125]. In the second step, adding the acceptance variables did not significantly improve the model [F(2,293), 0.07, p = .9287, R^2^ change = -0.21%]; personal acceptance and external acceptance did not significantly relate to stress scores [both p > .0125], thus contradicting the results from Cage et al. [38].

#### Do personal acceptance and external acceptance significantly relate to camouflaging?

In line with the procedures outlined in Perry et al. [60], we conducted a multiple regression analysis with camouflaging as the dependent variable, and external acceptance, personal acceptance, age, gender, age of diagnosis, and the level of autistic traits as predictors. In conflict with Perry et al. [60], the only significant relationship was between camouflaging and the level of autistic traits [t(294) = 8.02, p < .0001; all other p > .0125].

#### Does camouflaging significantly relate to anxiety?

As in Hull et al. [59], we conducted a hierarchical regression analysis with anxiety as the dependent variable. In step one, age and autistic traits were added into the model; in step two, camouflaging was added. The variables in step one (age and AQ) accounted for 2.30% of the variance, however, neither related to anxiety [p > .0125]. In step two, adding camouflaging significantly improved the model [F(1,302) = 20.84, p < .0001, R^2^ change = 6.01%]; in line with Hull et al., [59], those who camouflaged their autistic traits to a greater extent reported greater anxiety [t(302) = 4.57, p < .0001].

Additional exploratory analyses were conducted to evaluate the association between different types of external acceptance and depression, the associations between camouflaging and depression and stress, and to assess whether strengths of associations between our variables of interest differed across genders or country groups. These analyses are outside of the registered replications and are therefore reported in S3 File.

### Registered analyses: Comparing autism acceptance, camouflaging and mental health difficulties across countries

To compare participants’ experiences of autism acceptance, camouflaging, and levels of depression, anxiety and stress across countries, we conducted a multivariate Kruskal-Wallis test (since we were unable to establish univariate normality in each country across all six dependent variables). This analysis included a between-subjects factor *country*, and the dependent variables *external acceptance*, *personal acceptance*, *camouflaging*, *depression*, *anxiety*, and *stress*. Here we identified a significant main effect of country across all of these outcome variables [H(42) = 150.92, p < .0001]; for external acceptance [H(7) = 17.75, p = .0132], personal acceptance [H(7) = 23.86, p = .0012], camouflaging [H(7) = 15.63, p = .0287], depression [H(7) = 16.62, p = .0200], anxiety [H(7) = 29.01, p = .0001], and stress [H(7) = 25.73, p = .0006].

To unpack these effects, we next completed a univariate ANOVA for each variable which satisfied the conditions for parametric tests (external acceptance) and a Kruskal-Wallis test for each of the other variables (personal acceptance, camouflaging, depression, anxiety, and stress). In both cases, we used pairwise comparisons (t-tests for parametric analyses and Dunn’s tests for non-parametric analyses) with a Benjamini Hochberg False Discovery Rate correction (FDR; [112]) since this adjustment is more powerful than the Bonferroni-type adjustment methods and is recommended for scenarios in which a large number of hypotheses are simultaneously tested (e.g., 16–64 hypotheses) [112–117]. For the following analyses, there are 28 pairwise comparisons per outcome variable and therefore this method of adjustment is most suitable.

#### Do levels of autism acceptance vary across cultures?

**Fig 4:**
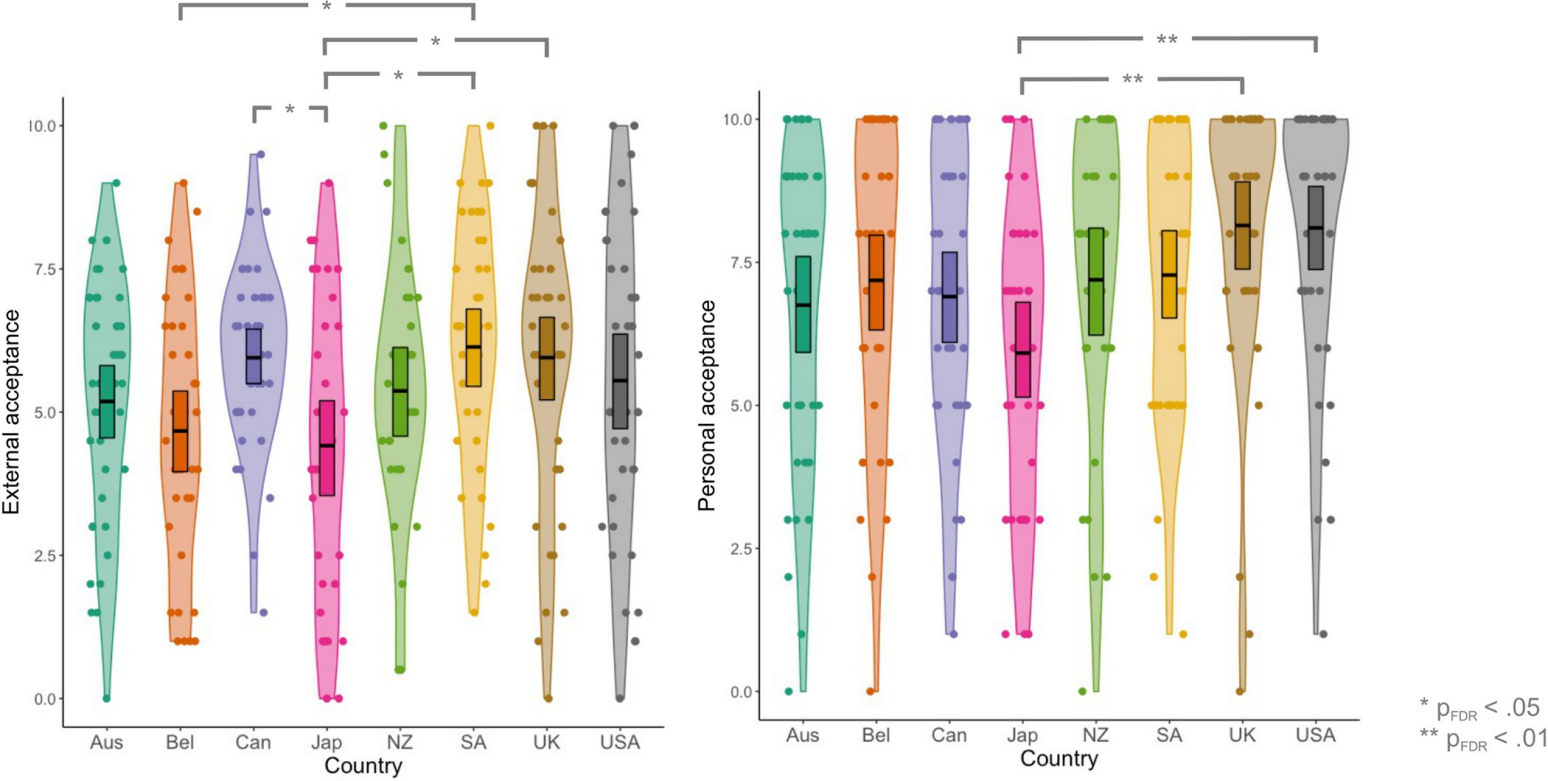
Levels of external acceptance (left) and personal acceptance (right) experienced by autistic individuals across Australia (Aus), Belgium (Bel), Canada (Can), Japan (Jap), New Zealand (NZ), South Africa (SA), the United Kingdom (UK), and the United States of America (USA).

Our analyses revealed that external acceptance was significantly lower in Japan [mean(standard error of the mean; SEM) = 4.41(0.39)] than in South Africa [mean(SEM) = 6.14(0.36); t(73) = -3.21, p_FDR_ = .0349], the UK [mean(SEM) = 5.95(0.35); t(75) = -2.67, p_FDR_ = .0349] and Canada [mean(SEM) = 5.95; t(73) = 3.18, p_FDR_ = .0349]. External acceptance was also lower in Belgium [mean(SEM) = 4.67(0.36)] than in South Africa [t(76) = -2.95, p_FDR_ = .0349; see Fig 4, left]. In addition, we identified that personal acceptance was significantly lower in Japan [mean(SEM) = 5.91(0.44)] than in the UK [mean(SEM) = 8.14(0.40); Z = -4.02, p_FDR_ = .0016] and USA [mean(SEM) = 8.10(0.41); Z = -3.83, p_FDR_ = .0018; see Fig 4, right]. There were no other significant differences in the level of external or personal acceptance across the countries studied. In sum, external acceptance is lowest in Japan and Belgium and highest in the UK, South Africa, and Canada; personal acceptance is lowest in Japan and highest in the UK and USA.

#### Does the degree of camouflaging vary across cultures?

**Fig 5:**
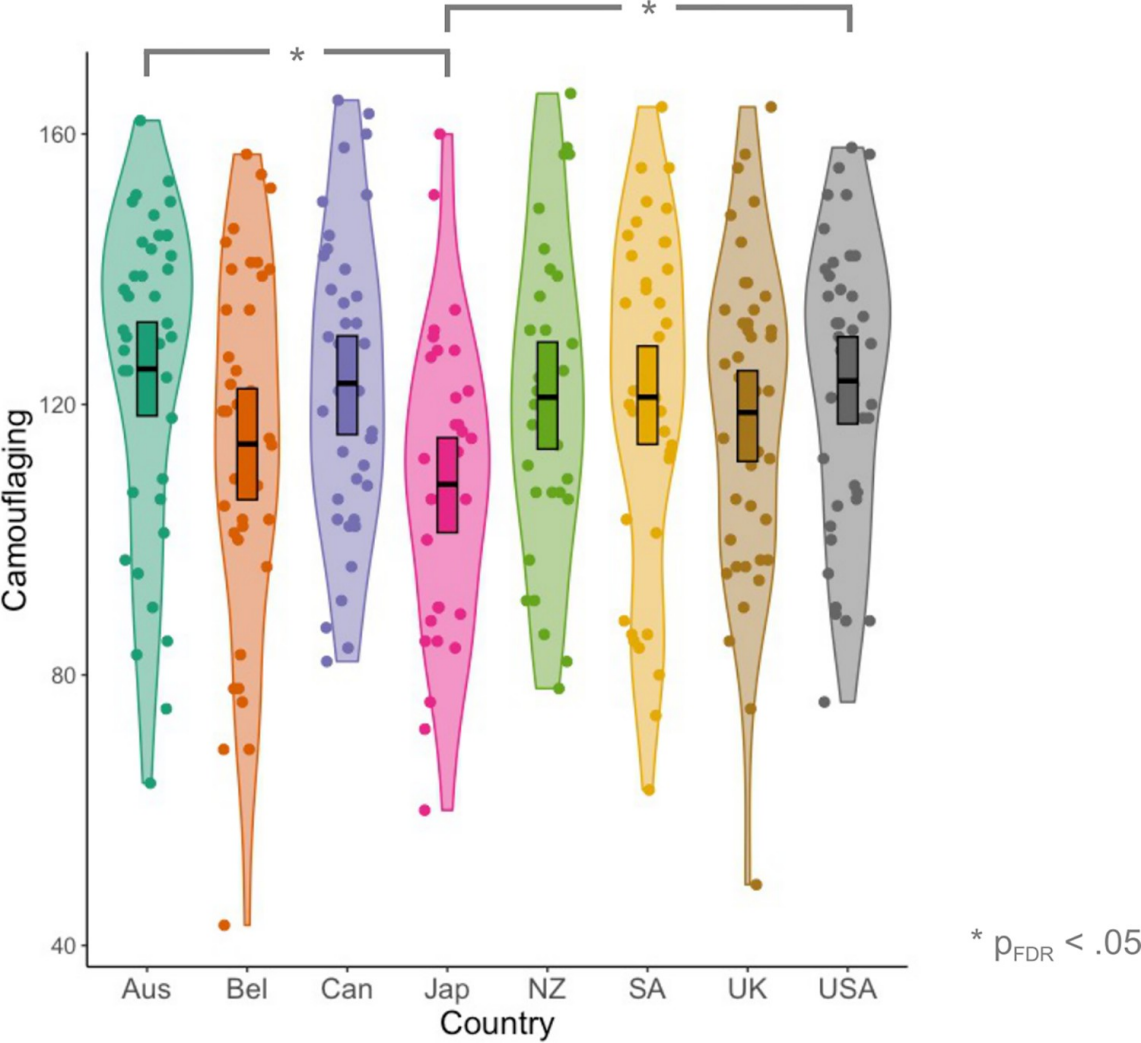
Levels of camouflaging across Australia (Aus), Belgium (Bel), Canada (Can), Japan (Jap), New Zealand (NZ), South Africa (SA), the United Kingdom (UK), and the United States of America (USA).

Our analysis also identified that camouflaging was significantly lower in Japan [mean(SEM) = 108.20(3.90)] than in Australia [mean(SEM) = 125.2(3.78); Z = 3.45, p_FDR_ = .0155] and the USA [mean(SEM) = 123.5(3.40); Z = -2.93, p_FDR_ = .0478; see Fig 5].

#### Do levels of mental health difficulties vary across cultures?

**Fig 6:**
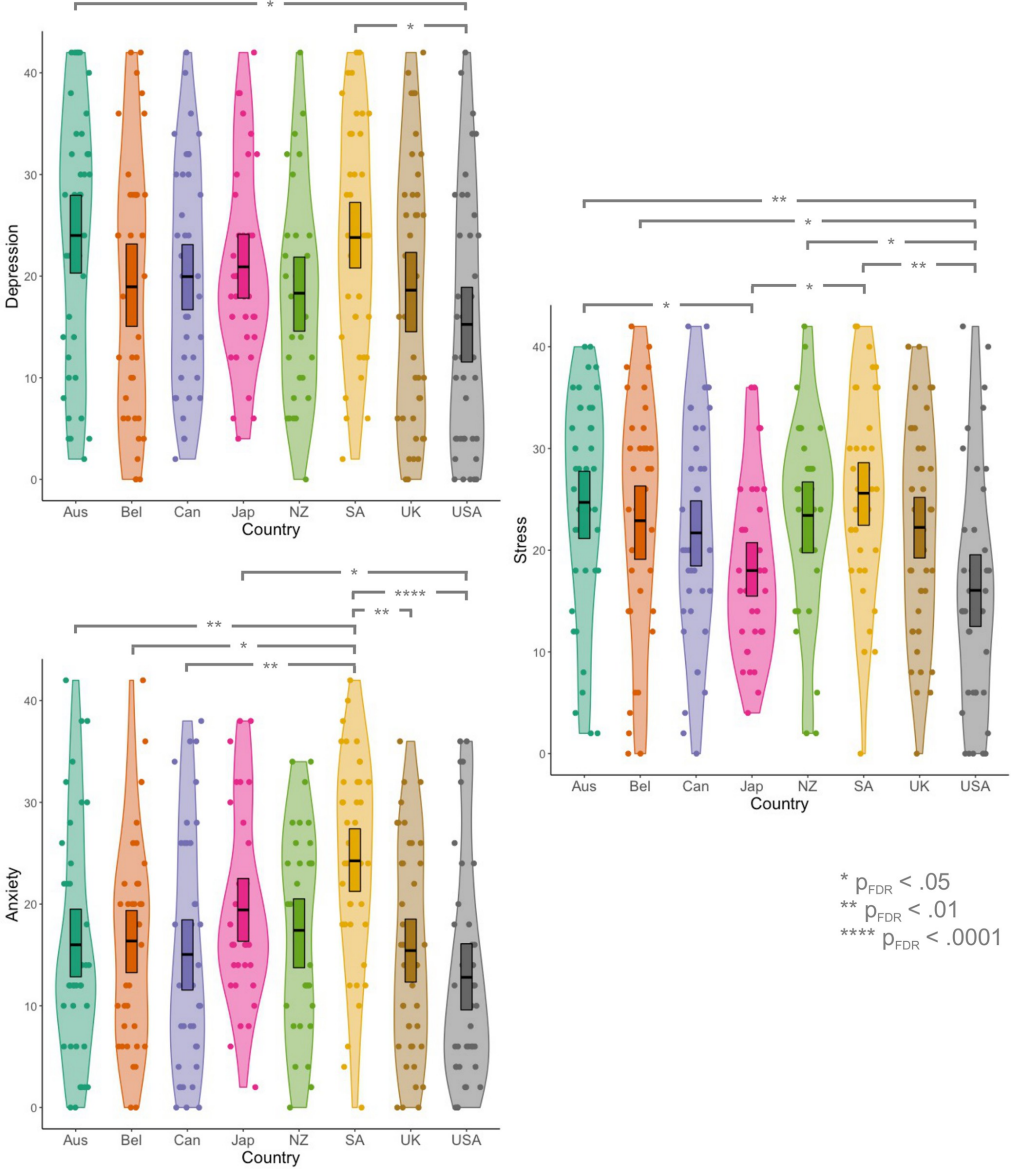
Levels of depression (top left), anxiety (bottom left), and stress (right) across Australia (Aus), Belgium (Bel), Canada (Can), Japan (Jap), New Zealand (NZ), South Africa (SA), the United Kingdom (UK), and the United States of America (USA).

Next, we aimed to compare the levels of depression, anxiety, and stress that the autistic participants experienced across each country. Our analysis identified that autistic individuals in South Africa [mean(SEM) = 23.80(1.85); Z = 3.25, p = .0163] and Australia [mean(SEM) = 24.00(1.85); Z = 3.27, p = .0299] experienced significantly higher levels of depression than those in the USA [mean(SEM) = 15.25(1.85); see Fig 6, top left].

Similarly, we identified that autistic people in South Africa [mean(SEM) = 24.25(1.65)] experienced significantly higher levels of anxiety than in the USA [mean(SEM) = 12.80(1.65); Z = 4.76, p_FDR_ < .0001], Canada [mean(SEM) = 15.05(1.65); Z = -3.78, p_FDR_ = .0022], the UK [mean(SEM) = 15.43(1.61); Z = 3.54, p_FDR_ = .0037], Australia [mean(SEM) = 16.00(1.65); Z = -3.39, p_FDR_ = .0048], and Belgium [mean(SEM) = 16.37(1.69); Z = -3.11, p_FDR_ = .0106]. In addition, the autistic individuals in Japan [mean(SEM) = 19.43(1.77)] experienced higher levels of anxiety than in the USA [Z = 2.89, p_FDR_ = .0177; see Fig 6, bottom left].

Finally, our analysis revealed that the autistic participants in the USA [mean(SEM) = 16.05(1.66)] experienced significantly lower levels of stress than those in South Africa [mean(SEM) = 25.60(1.66); Z = 3.85, p_FDR_ = .0033], Australia [mean(SEM) = 24.70(1.66); Z = 3.60, p_FDR_ = .0045], New Zealand [23.42(1.89); Z = 2.82, p_FDR_ = .0223], and Belgium [22.90(1.71); Z = 2.83, p_FDR_ = .0254]. In addition, autistic individuals in Japan [mean(SEM) = 18.00(1.78)] had significantly lower stress scores than in South Africa [Z = -3.21, p_FDR_ = .0122] and Australia [Z = 2.97. p_FDR_ = .0208; see Fig 6, right].

In sum, there are different profiles of mental health difficulties across each of the countries studied: autistic individuals in South Africa experience the highest levels of depression, anxiety, and stress, while those in the USA experience the lowest; those in Australia experience difficulties with depression and stress but have comparable levels of anxiety to the other countries studied; those in Japan have elevated levels of anxiety, but low levels of stress.

### Exploratory analyses: Are there differences in acceptance, camouflaging and mental health difficulties after controlling for relevant individual differences?

To assess the contribution of various individual differences to acceptance, camouflaging and mental health, we conducted a) bivariate correlations between our outcome variables and continuous individual difference variables (e.g., age, age of diagnosis, autistic traits, alexithymic traits), and b) Kruskal-Wallis tests comparing our outcome variables across categorical groups (e.g., gender, sexuality, level of education, income, etc.). This identified that age, age of diagnosis, gender, the level of autistic traits, the level of alexithymic traits, sexuality, level of education, current verbal level, and presence of co-occurring conditions contributed to at least one of the outcome variables (see S4 File).

Following this, we ran a series of parametric ANCOVAs (for external acceptance) and ranked ANCOVAs (for variables where parametric assumptions were not met: personal acceptance, camouflaging, depression, anxiety and stress) predicting our outcome variables with country group after controlling for these relevant covariates. This allowed us to verify that any differences found across countries in acceptance, camouflaging, and mental health were not underpinned by differences in demographic factors across groups (e.g., age, gender, etc.). These analyses demonstrated that external acceptance, personal acceptance, depression, anxiety, and stress significantly differed across countries, after controlling for age, age of diagnosis, gender, the level of autistic traits, the level of alexithymic traits, sexuality, level of education, verbal ability, and the presence of co-occurring conditions. By contrast, the main effect of country on camouflaging became non-significant after controlling for these relevant demographic factors.

In our ANCOVA with external acceptance, the significant main effect of country remained [F(7,273) = 3.94, p = .0004], after controlling for the covariates. This analysis also revealed that higher age [F(1,273 = -15.27, p = .0001] and the presence of co-occurring conditions [F(2,273) = 8.47, p = .0003] were associated with lower external acceptance. In addition, verbal level had a significant effect on external acceptance [F(2,273) = 3.39, p = .0350]: non-verbal individuals [mean(SEM) = 7.17(0.51)] experienced higher levels of external acceptance than minimally verbal [mean(SEM) = 5.31(0.06)] and verbal [mean(SEM) = 5.43(0.01)] individuals. Similarly, in our ranked ANCOVA with personal acceptance, the significant main effect of country remained [F(7,274) = 3.20, p = .0028] after controlling for the covariates. Additionally, this analysis revealed that higher levels of autistic traits [F(1,274) = 4.61, p = .0327], lower levels of alexithymic traits [F(1,274) = -4.10, p = .0439], and an absence of co-occurring conditions [F(2,274) = 3.23, p = .0412] were associated with greater levels of personal acceptance.

In our ranked ANCOVA with camouflaging, we identified that the significant effect of country group disappeared [F(7,274) = 0.68, p = .6908] after controlling for the covariates. This analysis also identified that the level of autistic traits [F(1,274) = 14.80, p = .0002] and the level of alexithymic traits [F(1,274) = 3.88, p = .0497] were significantly related to camouflaging: those higher in autistic and alexithymic traits camouflaged their autistic traits more. Finally, we also found that camouflaging differed across genders [F(5,274) = 2.42, p = .0359]: autistic individuals that identified as non-binary/Third gender [mean(SEM) = 132.8] and women [mean(SEM) = 123.8] camouflaged more than men [mean(SEM) = 109.9].

Next, our ranked ANCOVA with depression identified that the main effect of country group remained significant [F(7,274) = 2.50 p = .0167] after controlling for the covariates. In addition, the level of alexithymic traits [F(1,274) = 11.31 p = .0009] and the presence of co-occurring conditions [F(2,274) = 7.37, p = .0008] significantly related to levels of depression: those higher in alexithymic traits and/or with co-occurring conditions typically experienced higher levels of depression.

In our ranked ANCOVA with anxiety, we identified that the significant main effect of country remained [F(7,274) = 4.17 p = .0002] after controlling for the covariates. Moreover, the level of alexithymic traits [F(1,274) = 24.83, p < .0001] and the presence of co-occurring conditions [F(2,274) = 5.52, p = .0045] significantly related to levels of anxiety: those higher in alexithymic traits and/or with co-occurring conditions experienced higher levels of anxiety. Finally, anxiety differed as a function of verbal level [F(2,274) = 9.28, p = .0001]: non-verbal individuals experienced the highest levels of anxiety [mean(SEM) = 29.33], followed by minimally verbal individuals [mean(SEM) = 25.53], followed by verbal [mean(SEM) = 15.77] individuals.

Finally, our ANCOVA with stress identified that the main effect of country group remained significant [F(7,274) = 2.18, p = .0361] after controlling for the covariates. In addition, the level of alexithymic traits [F(1,274) = 34.39, p < .0001] and the presence of co-occurring conditions [F(2,274) = 5.08, p = .0068] related to levels of stress: those higher in alexithymic traits and/or with co-occurring conditions experienced higher stress.

## Discussion

The current study comprises the first ever cross-cultural investigation of autistic individuals’ experiences of autism acceptance, camouflaging, and mental health difficulties. There were two primary aims of this study: (1) to determine whether significant relationships between autism acceptance, camouflaging, and mental health difficulties replicate in a more diverse cross-cultural sample, and (2) to compare these variables across countries. With respect to the former, our results partially replicate those found previously, in a more diverse cross-cultural sample of autistic adults. That is, in line with Hull et al., [59] we found that anxiety was associated with camouflaging across all countries studied (Australia, Belgium, Canada, Japan, New Zealand, South Africa, UK, and USA): those who camouflaged their autistic traits to a greater extent experienced elevated levels of anxiety (along with heightened depression and stress; see S3 File). Moreover, we partially replicated the findings from Cage et al. [38]: whilst depression was (negatively) associated with both personal acceptance and external acceptance (though note that these variables explain similar portions of variance), stress was not associated with external acceptance (in contrast to Cage et al. [38]). Finally, in conflict with Perry et al. [60], the extent to which individuals camouflaged their autistic traits did not relate to the degree of personal or external acceptance.

The finding that camouflaging one’s autistic traits is associated with higher levels of anxiety adds to a growing literature suggesting a link between camouflaging and mental health [26, 38, 54–58]. This literature proposes several potential mechanisms that may underlie this association. One argument posits that regularly pretending not to be autistic erodes one’s sense of identity [39], which is known to be crucial for mental health [118–122]. Additionally, since camouflaging is reported to be exhausting [39, 56], another argument is that it depletes one’s mental resources, leading to poorer mental health outcomes. Finally, it is thought that camouflaging can result in the needs of the individual being misunderstood or overlooked entirely [54], thereby depriving them of the support they need, and further contributing to diminished mental wellbeing. Due to the substantive qualitative research on potential mechanisms, we have suggested that camouflaging one’s autistic traits may contribute to higher levels of anxiety, however, it is important to note that the opposite direction of causality is also plausible (as this study is cross-sectional). For example, since highly anxious individuals have increased threat perception (e.g., [123–125]) it may be that these individuals camouflage their autistic traits to mitigate this potential threat. If this argument is true, we would expect threat perception to mediate the relationship between anxiety and camouflaging (though this is yet to be tested). Another possibility is that there is a bidirectional or cyclical relationship between these variables such that camouflaging leads to elevated anxiety, which in turn leads to even higher camouflaging, and so on. Further research is necessary to verify the direction of the relationships between these variables. Longitudinal research, which examines how relationships between acceptance, camouflaging, and mental health change across time, will be particularly beneficial for establishing directionality. Additionally, research that involves causal manipulation, for example studies assessing the impact of autism awareness campaigns, on levels of perceived acceptance, camouflaging, and mental health difficulties, will be useful for establishing causality between these variables.

There are a number of potential explanations for why we did not fully replicate the results from Cage et al. [38] and Perry et al. [60]. Firstly, since the sample in the current study was larger than those recruited previously (here N = 306; Cage et al., N = 111; Perry et al., N = 233), and larger samples result in more precise parameter estimates [126], the effect sizes in previous studies may have been inflated to some extent. Another possibility is that the discrepancy in findings results from differences in mean levels of acceptance and camouflaging across samples. Participants in the current study experienced higher levels of external acceptance [mean = 5.43] and lower levels of camouflaging [mean = 119.56] than in Cage et al., [external acceptance mean = 4.86] and Perry et al., [camouflaging mean = 126.65]. Therefore, our sample can be said to have less severe experiences of stigma and camouflaging than previous samples and, as such, associations found previously may be driven by those with ‘more severe’ experiences. Further research is necessary to test whether these associations hold in samples who have experienced higher levels of stigma and camouflaging.

Another explanation for why we did not replicate the results from Perry et al. [60], specifically, is that we used a different scale to assess stigma/acceptance. In our study, we used the autism acceptance questions from Cage et al., [38], whilst Perry et al., [60] used an adapted version of the Stigma Consciousness Scale, which assesses *awareness* of stigmatized status. Since this scale involves five items on a four-point Likert scale (in comparison to just two items for external acceptance here), it may be more sensitive to individual differences in perceived stigma/acceptance than our measure, which may explain why we did not detect a significant association between acceptance and camouflaging. An alternative explanation is that there is conceptual overlap in the items of Stigma Consciousness Scale and the CAT-Q, that is not present between the CAT-Q and Cage’s autism acceptance questions. For example, the question “I almost never think about the fact I am autistic when I’m around others” in the Stigma Consciousness Scale is similar to the “I always think about the impression I make on other people” and “I am always aware of the impression I make on other people” items from the CAT-Q. Although these questions are distinct to some degree–the former concerns autism specifically while the latter two concern the impression one makes more generally (which may be affected by other personal characteristics)–there is some conceptual overlap between them. This overlap may augment the strength of the association between stigma and camouflaging, resulting in a significant effect (as in [60]). Thus, further research, using measures that assess fully distinct concepts, is necessary to understand the extent of the association between acceptance and camouflaging.

Our results highlight that there are differences in levels of autism acceptance, camouflaging, and mental health difficulties across country groups. When interpreting such cross-cultural differences, it is important to consider the equivalence of samples across groups, which can be influenced by factors such as sampling, accessibility of autism diagnoses, and differences in demographic factors across countries (e.g., age, age of diagnosis, level of autistic traits, etc.). Notably, we find that the majority of the cross-cultural differences remain even after controlling for relevant factors that may vary across each of our countries (e.g., age, age of diagnosis, gender, the level of autistic traits, the level of alexithymic traits, sexuality, level of education, verbal ability, and presence of co-occurring conditions). Therefore, our results provide convincing evidence for genuine cross-cultural variation in these factors, independent of variables that may distinguish participants across country groups.

In line with our hypothesis, and previous studies identifying cross-cultural variation in attitudes (held by non-autistic people) towards autistic people [68–70], our results show cross-cultural differences in levels of acceptance *experienced by autistic people*, even after controlling for relevant covariates. Specifically, we found that individuals in Japan experienced lower levels of both external and personal acceptance, while individuals in Belgium experienced lower levels of external acceptance than at least one of the other countries studied. There are many factors that could be driving these differences in levels of acceptance across countries, such as varying levels of autism-related knowledge or differences in cultural orientation. With respect to the former, a growing body of research suggests that those with poorer awareness of autism typically exhibit higher levels of stigma towards autistic people [68–70, 127–130]. Hence, it could be the case that individuals in Belgium and Japan have reduced knowledge about autism (relative to other countries) and therefore display greater stigma towards autistic people–though research is necessary to confirm this. If this is true, it would be advisable to employ autism knowledge interventions, like those conducted previously [70, 83, 131–133], in Belgium and Japan to increase awareness about, and decrease stigma towards, autism. Although this is an appropriate avenue for future research, it is unlikely that soley differences in knowledge can explain cross-cultural differences in stigma: recent research revealed that differences between Japan and USA in stigma remain even after controlling for autism-related knowledge [70]. As such, it is important to consider other factors that may contribute to autism-related stigma.

Another potential contributing factor is cultural orientation. It is reasonable to assume that stigma associated with autism–a condition characterised by difficulties fitting into a neurotypical world not built for them–might be more pronounced in a society with greater emphasis on shared group norms (i.e., collectivist cultures) than in one that prioritizes independence (i.e., individualist cultures), as being unique may be perceived as a threat in interdependent cultures [70]. Concurrently, stigma may be higher in societies that place greater emphasis on hierarchy (i.e., vertical cultures) than in those that emphasize equality between its members (i.e., horizontal cultures). Indeed, a growing body of evidence suggests that autism-related stigma is higher in collectivist than individualistic cultures (e.g., Japan: [70]; Lebanon: [68, 69]; South Korea: [127]), and that higher verticality is linked to greater stigma towards autistic and neurodivergent people [68, 134]. Notably, differences in cultural orientation may underpin the cross-country variation in acceptance documented here. In the current study, external acceptance was lowest in Japan followed by Belgium; notably Japan is lowest in individualism (Japan = 46; South Africa = 65; Belgium = 75; New Zealand = 79; Canada = 80; UK = 89; Australia = 90; USA = 91 [86, 87]), and both Belgium and Japan are higher in verticality (Belgium = 65; Japan = 54; South Africa = 49; USA = 40; Canada = 39; Australia = 38; UK = 35; New Zealand = 22 [86, 87]), than the other countries studied. Further research is necessary to confirm whether differences in cultural orientation underpin cross-country variation in levels of acceptance. Such research could also attempt to study other aspects of cultural orientation, such as masculinity, uncertainty avoidance, long term orientation, and indulgence [86, 87, 135, 136], thus moving beyond the binary categorisation of culture that is often seen in the literature (i.e., individualism-collectivism; see [137] for a critique). In addition, rather than assuming that all individuals within each country conform to their cultural norm (e.g., all individuals in the USA are individualists), future studies could index the orientiation of each individual participant (e.g., with scales like the Cultural Value Orientation Scale [138]), thus allowing for intra-cultural variation in these factors. Finally, further research could aim to determine the *relative* contributions of cultural orientation, knowledge and other potentially relevant factors (e.g., openness to experience, contact with autistic people; see S5 File for prevalence rates across countries [68, 127]) to autism-related stigma. Such work has the potential to illuminate routes to increased acceptance of autism.

In addition, we identified significant differences in levels of camouflaging across countries, with Japanese individuals scoring the lowest, though notably these differences did not remain after controlling for relevant covariates (e.g., age, age of diagnosis, gender, sexuality, etc.). Further research is necessary to confirm whether rates of camouflaging are lower in Japanese autistic adults; indeed one other large study recently identified even lower camouflaging scores for this group than documented here (see [96]). If this is confirmed, studies should explore potential influencing factors. For example, it could that autistic individuals in Japan *perceive* that they are camouflaging their autistic traits less because they are acustomed to adapting other parts of their identity in order to minimise deviation from shared group norms. Alternatively, assuming that the aim of camouflaging is to reach what would be considered a “typical” level of autistic traits, one explanation is that individuals in Japan have to camouflage less (than in other countries) to reach such a threshold. This is plausible since the levels of autistic traits in large general population samples appear to be higher in Japan (means = 21.32–23.29 [139]) than in Australia and the USA (i.e., the countries where we found differences; mean = 16.03–16.72 [140, 141]). Hence, autistic individuals in Japan may not need to camouflage their autistic traits as much in order to ‘fit into’ their society. Relatedly, the extent to which autistic individuals engage in camouflaging will also depend on the *specific* environments they find themselves in, and importantly these environments may vary across cultures. For example, in certain countries, higher proportions of autistic adults may be unemployed or in supported employment, where there may be less pressure to camouflage one’s autistic traits. Conversely, in unsupported employment settings, there may be greater pressure to conform to neurotypical norms. This explanation is plausible since there are higher rates of unemployment and supported employment, and lower rates of unsupported employment, amongst autistic adults in Japan [142] than Australia and the USA (i.e., where we found group differences [143, 144]). As such, the autistic participants in Japan may not need to camouflage their autistic traits as much to fit in to their *specific* workplace environments (and therefore may report lower camouflaging). Further research is neccesary to confirm (1) whether rates of camouflaging are lower in Japan, and (2) the mechanisms underpinning such differences.

Finally, in the current study, we identified different profiles of mental health difficulties across countries. Specifically, we found that autistic individuals in South Africa experienced the highest mental health burden, displaying elevated levels of depression, anxiety, and stress, while those in the USA experienced the lowest. Notably, this increased mental health burden in South Africa may not be unique to the autistic population but rather reflect increased mental health difficulties in the general population. Future studies employing well-matched non-autistic samples (e.g., as in [44]) are necessary to test whether these cross-cultural differences are unique to the autistic population. Nevertheless, there are several potential explanations for these cross-cultural differences in mental health difficulties. One idea is that there are systemic and attitudinal barriers to accessing support in certain countries [79–81], thus leading to greater prevalence and severity of mental health difficulties. This explanation is particularly plausible given that 47.2% of adults with a mental illness in the USA accessed mental health services in the past year [145] in comparison to just 25.5% of individuals in South Africa [146]. This discrepancy could be due to several systemic barriers: insufficient funding, a lack of human resources and facilities, a scarcity of child and adolescent services, and inconsistent availability of medication all pervade South African health services [147]. Concurrently, a lack of awareness about, or stigma towards, mental illness [147, 148], low perceived need for treatment, or perceptions that the support will be ineffective may also contribute to this discrepancy [148]. Consequently, as highlighted by the South African Human Rights Commission [147], it is necessary to dismantle systemic barriers (e.g., design services for children and adolescents) as well as attitudinal barriers (e.g., by introducing stigma reduction programs in schools to increase willingness to access treatment; see [147] for full recommendations) to mental health support, thereby reducing the levels of mental health difficulties in both the general and autistic populations.

### Limitations

In the current study we have accomplished our aim of recruiting a more diverse, cross-cultural, sample of autistic adults. Here we have tested participants that speak three different native languages, across eight countries (including one middle-income country; South Africa) spanning five continents (Africa, Asia, Europe, North America, and Oceania). In addition, based on comparisons to large populations, the sample is representative of the broader autistic community in terms of sexuality (current study: 58.91% of individuals that reported their sexuality were heterosexual, 20.00% bisexual, 10.18% lesbian, gay or homosexual, and 10.91% other; UK sample in Weir, Allison & Baron-Cohen [149] 63.03% heterosexual, 13.63% bisexual, 7.58% lesbian, gay or homosexual, 15.76% Other), level of autistic traits (current study mean = 34.01; Ruzich et al. [150] mean = 35.19), and level of alexithymic traits (current study: 52.94% alexithymic; Kinnaird, Stewart & Tchanturia [32]: 49.93% alexithymic). Nevertheless, there are a number of shortcomings of our sample with respect to generalizability. Notably, the respondents in our survey were predominantly white (54.3%), verbal (85.6%), highly educated individuals (48.0% with university education), from developed individualistic countries (e.g., Australia, Belgium, Canada, New Zealand, UK, USA). As such, our results may not represent racially or ethnically minoritized individuals, those who are non-verbal or minimally verbal (here 14.4%, estimated to be 30.0% of autistic adults; [151, 152]), those with lower levels of education or intellectual disabilities, and those from different cultural backgrounds. In addition, although the proportion of non-binary individuals in our sample is comparable to that of the broader autistic population (current study: 8.5%; previous studies: approximately 4–12% [153, 154]), autistic females may be over-represented, and autistic males under-represented, in the current sample. While recent estimates suggest that the relative proportion of autistic males to females is around 3:1 [155], there are similar numbers of autistic males (42.8%) and females (43.5%) in our sample. Therefore, our findings may not be representative of the broader, predominantly male, autistic population. Finally, participants in the current study were required to have access to a computer/mobile device and internet; consequently, we encourage future studies to find ways to dismantle such barriers to inclusion (e.g., sending written versions of surveys to participants, participants completing the survey over the phone, etc.).

## Conclusion

In the current study, we aimed to (1) determine whether significant relationships between autism acceptance, camouflaging, and mental health difficulties replicate in a more diverse cross-cultural sample of autistic adults, and (2) compare these variables across cultures. With respect to the former, we partially replicated previous results: external acceptance and personal acceptance were associated with lower levels of depression but not camouflaging or stress, and higher camouflaging was associated with elevated levels of anxiety. With respect to the latter, we found significant differences across countries in external acceptance, personal acceptance, depression, anxiety, and stress, even after controlling for relevant covariates. These findings have significant implications, identifying priority regions for anti-stigma interventions and highlighting countries where greater mental health support is needed.

**Registered report protocol:** Keating, C. T., Hickman, L., Geelhand, P., Takahashi, T., Leung, J., Schuster, B.,… & Sowden, S. (2021). Global perspectives on autism acceptance, camouflaging behaviours and mental health in autism spectrum disorder: A registered report protocol. *Plos one*, *16*(12), e0261774. https://doi.org/10.1371/journal.pone.0261774.

## Supporting information

S1 File Additional participant demographics. Information regarding participant’s levels of education, income, current verbal level, childhood verbal level, sexuality, autism diagnoses, and age of diagnoses, race and ethnic groups.
(DOCX)

S2 File Item and factor analyses on translated versions of the scales. Item and factor analyses on the translated autism acceptance questions and CAT-Q.
(DOCX)

S3 File Exploratory regression analyses. Exploratory regression analyses assessing the relationships between our variables of interest.
(DOCX)

S4 File Determining the relationships between our variables of interest and control variables. The association between age, age of diagnosis, autistic traits, alexithymic traits, gender, sexuality, level of education, current verbal level, and the presence of co-occurring conditions with our variables of interest.
(DOCX)

S5 File Estimated prevalence of autism across countries. (DOCX)
