# Supplementary material for: Cross-cultural variation in experiences of acceptance, camouflaging and mental health difficulties in autism: A registered report
Source: PLoS One. 2024 Mar 20;19(3):e0299824. doi: 10.1371/journal.pone.0299824 (PMC10954134; doi:10.1371/journal.pone.0299824)
Supplement: S1 File — Information regarding participant’s levels of education, income, current verbal level, childhood verbal level, sexuality, autism diagnoses, and age of diagnoses, race and ethnic groups. (DOCX) [file pone.0299824.s001.docx]

**Supporting Information**

**S1 File - Participant demographics**

**Table S1.** Participants’ level of education, income, current verbal level, and childhood verbal level across each country group.

| Country | Level of education | Income (in $USD) | Current verbal level | Childhood verbal level |
| --- | --- | --- | --- | --- |
| Australia  (N = 40) | 1- Primary education,  16 - Lower secondary education  5 - Upper secondary education  4 - Post-secondary non-tertiary education  0 - Short-cycle tertiary education  9 - Bachelor’s or equivalent level  3 - Master’s or equivalent level  2 - Doctoral or equivalent level | 2 - Choose not to disclose  9 - Up to $9,999  13 - $10,000-$19,999  3 - $20,000-$29,000  3 - $30,000-$39,000  1- $40,000-$49,000  3 - $50,000-$59,000  3 - $60,000-$69,000  3 - $70,000-$79,000  0 - $80,000-$89,000  0 - $90,000-$99,000  0 - $100,000-$109,000  0 - Over $110,000 | 36 - Verbal  4 - Minimally verbal  0 - Non-verbal | 32 - Verbal  8 - Minimally verbal  0 - Non-verbal |
| Belgium  (N = 38) | 0 - Primary education,  0 - Lower secondary education  18 - Upper secondary education  0 - Post-secondary non-tertiary education  0 - Short-cycle tertiary education  2 - Bachelor’s or equivalent level  17 - Master’s or equivalent level  1 - Doctoral or equivalent level | 2 - Choose not to disclose 6 - Up to $9,999  12 - $10,000-$19,999  6 - $20,000-$29,000  5 - $30,000-$39,000  3 - $40,000-$49,000  2 - $50,000-$59,000  0 - $60,000-$69,000  2 - $70,000-$79,000  0 - $80,000-$89,000  0 - $90,000-$99,000  0 - $100,000-$109,000  0 - Over $110,000 | 35 - Verbal  3 - Minimally verbal  0 - Non-verbal | 30 - Verbal  8 - Minimally verbal  0 - Non-verbal |
| Canada  (N = 40) | 1 - Primary education,  14 - Lower secondary education  3 - Upper secondary education  2 - Post-secondary non-tertiary education  0 - Short-cycle tertiary education  15 - Bachelor’s or equivalent level  4 - Master’s or equivalent level  1 - Doctoral or equivalent level | 3 - Choose not to disclose  12 - Up to $9,999  10 - $10,000-$19,999  2 - $20,000-$29,000  2 - $30,000-$39,000  2 - $40,000-$49,000  2 - $50,000-$59,000  1 - $60,000-$69,000  2 - $70,000-$79,000  1 - $80,000-$89,000  1 - $90,000-$99,000  2 - $100,000-$109,000  0 - Over $110,000 | 35 - Verbal  5 - Minimally verbal  0 - Non-verbal | 28 - Verbal  11 - Minimally verbal  1 - Non-verbal |
| Japan  (N = 35) | 0 - Primary education,  1 - Lower secondary education  15 - Upper secondary education  3 - Post-secondary non-tertiary education  0 - Short-cycle tertiary education  15 - Bachelor’s or equivalent level  1 - Master’s or equivalent level  0 - Doctoral or equivalent level | 2 - Choose not to disclose  22 - Up to $9,999  7 - $10,000-$19,999  4 - $20,000-$29,000  0 - $30,000-$39,000  0 - $40,000-$49,000  0 - $50,000-$59,000  0 - $60,000-$69,000  0 - $70,000-$79,000  0 - $80,000-$89,000  0 - $90,000-$99,000  0 - $100,000-$109,000  0 - Over $110,000 | 35 – Verbal  0 – Minimally verbal  0 – Non-verbal | 30 - Verbal  5 - Minimally verbal  0 - Non-verbal |
| New Zealand  (N = 31) | 0 - Primary education,  2 - Lower secondary education  6 - Upper secondary education  8 - Post-secondary non-tertiary education  1 - Short-cycle tertiary education  9 - Bachelor’s or equivalent level  5 - Master’s or equivalent level  0 - Doctoral or equivalent level | 3 - Choose not to disclose  4 - Up to $9,999  7 - $10,000-$19,999  5 - $20,000-$29,000  5 - $30,000-$39,000  3 - $40,000-$49,000  1 - $50,000-$59,000  2 - $60,000-$69,000  0- $70,000-$79,000  0- $80,000-$89,000  1 - $90,000-$99,000  0 - $100,000-$109,000  0 - Over $110,000 | 28 - Verbal  3 - Minimally verbal  0 - Non-verbal | 20 - Verbal  6 - Minimally verbal  5 - Non-verbal |
| South Africa  (N = 40) | 0 - Primary education,  13 - Lower secondary education  4 - Upper secondary education  7 - Post-secondary non-tertiary education  0 - Short-cycle tertiary education  13 - Bachelor’s or equivalent level  3 - Master’s or equivalent level  0 - Doctoral or equivalent level | 3 - Choose not to disclose  24 - Up to $9,999  7 - $10,000-$19,999  2 - $20,000-$29,000  2 - $30,000-$39,000  2 - $40,000-$49,000  1 - $50,000-$59,000  1 - $60,000-$69,000  0 - $70,000-$79,000  0 - $80,000-$89,000  0 - $90,000-$99,000  0 - $100,000-$109,000  0 - Over $110,000 | 19 - Verbal  18 - Minimally verbal  3 - Non-verbal | 14 - Verbal  18 - Minimally verbal  8 - Non-verbal |
| UK  (N = 42) | 0 - Primary education,  4 - Lower secondary education  9 - Upper secondary education  4 - Post-secondary non-tertiary education  1 - Short-cycle tertiary education  13 - Bachelor’s or equivalent level  10 - Master’s or equivalent level  1 - Doctoral or equivalent level | 2 - Choose not to disclose  14 - Up to $9,999  8 - $10,000-$19,999  7 - $20,000-$29,000  5 - $30,000-$39,000  2 - $40,000-$49,000  1 - $50,000-$59,000  2 - $60,000-$69,000  0 - $70,000-$79,000  1 - $80,000-$89,000  0 - $90,000-$99,000  0 - $100,000-$109,000  0 - Over $110,000 | 39 - Verbal  3 - Minimally verbal  0 - Non-verbal | 30 - Verbal  9 - Minimally verbal  3 - Non-verbal |
| USA  (N = 40) | 0 - Primary education,  6 - Lower secondary education  6 - Upper secondary education  5 - Post-secondary non-tertiary education  0 - Short-cycle tertiary education  21 - Bachelor’s or equivalent level  2 - Master’s or equivalent level  0 - Doctoral or equivalent level | 0 - Choose not to disclose  11 - Up to $9,999  8 - $10,000-$19,999  3 - $20,000-$29,000  2 - $30,000-$39,000  7 - $40,000-$49,000  1- $50,000-$59,000  3 - $60,000-$69,000  1 - $70,000-$79,000  0 - $80,000-$89,000  0 - $90,000-$99,000  1 - $100,000-$109,000  3 - Over $110,000 | 35 - Verbal  5 - Minimally verbal  0 - Non-verbal | 28 - Verbal  11 - Minimally verbal  1 - Non-verbal |

**Table S2.** Participants’ sexuality, autism diagnoses, and age of diagnoses across each country group.

| Country | Sexuality | Autism diagnosis | Age of diagnosis |
| --- | --- | --- | --- |
| Australia  (N = 40) | 18 - Straight or heterosexual  10 - Bisexual  4 - Lesbian, gay or homosexual  3 - Queer  1 - Asexual/Aromantic/Questioning  1 - Demisexual/Asexual/Questioning  2 - I don’t know  1 - Choose not to disclose | 14 - Asperger’s syndrome  23 - Autism Spectrum Disorder  2 - Classic Autism/Autistic Disorder  1 - Choose not to disclose | 22.14(11.20) |
| Belgium  (N = 38) | 23 - Straight or heterosexual  5 - Bisexual  3 - Lesbian, gay or homosexual  2 - Asexual  4 - I don’t know  1 - Choose not to disclose | 19 - Asperger’s syndrome  13 - Autism Spectrum Disorder  4 - Classic Autism/Autistic Disorder  2 - I don’t know | 35.18(11.31) |
| Canada  (N = 40) | 16 - Straight or heterosexual  9 - Bisexual  7 - Lesbian, gay or homosexual  3 - Asexual  2 - Pansexual  2 - I don’t know  1 - Choose not to disclose | 19 - Asperger’s syndrome  15 - Autism Spectrum Disorder  3 - Classic Autism/Autistic Disorder  1 - PDD-NOS  2 - Choose not to disclose | 19.40(12.17) |
| Japan  (N = 35) | 22 - Straight or heterosexual  1 - Bisexual  10 - I don’t know  2 - Choose not to disclose | 11 - Asperger’s syndrome  17 - Autism Spectrum Disorder  5 - PDD-NOS  2 - I don’t know | 20.51(9.45) |
| New Zealand  (N = 31) | 15 - Straight or heterosexual  8 - Bisexual  3 - Lesbian, gay or homosexual  3 - Asexual  1 - Abrosexual  1 - I don’t know | 11 - Asperger’s syndrome  15 - Autism Spectrum Disorder  5 - Classic Autism/Autistic Disorder | 23.65(12.40) |
| South Africa  (N = 40) | 28 - Straight or heterosexual  5 - Bisexual  5 - Lesbian, gay or homosexual  1 - Pansexual  1 - Choose not to disclose | 6 - Asperger’s syndrome  13 - Autism Spectrum Disorder  9 - Classic Autism/Autistic Disorder  5 - Childhood disintegrative disorder  1 - PDD-NOS  6 - Choose not to disclose | 13.30(8.61) |
| UK  (N = 42) | 21 - Straight or heterosexual  8 - Bisexual  4 - Lesbian, gay or homosexual  4 - Asexual  1 - Pansexual  1 - Panromantic  2 - I don’t know  1 - Choose not to disclose | 16 - Asperger’s syndrome  21 - Autism Spectrum Disorder  4 - Classic Autism/Autistic Disorder  1 - Choose not to disclose | 23.76(12.93) |
| USA  (N = 40) | 19 - Straight or heterosexual  9 - Bisexual  2 - Lesbian, gay or homosexual  5 - Asexual  1 - Asexual/Panromantic  4 - I don’t know | 12 - Asperger’s syndrome  24 - Autism Spectrum Disorder  2 - Classic Autism/Autistic Disorder  1 - Childhood disintegrative disorder  1 - Social Communication Disorder | 18.76(12.96) |

**Table S3.** Participants’ race information.

| Racial Group | N | Percentage of sample (%) |
| --- | --- | --- |
| Asian | 45 | 14.71% |
| Black | 33 | 10.78% |
| Hispanic/Latinx | 5 | 1.63% |
| White | 166 | 54.25% |
| Mixed/Multiple Racial Groups | 17 | 5.56% |
| Not disclosed | 40 | 13.07% |
| Total | 306 | 100% |

**Table S4.** Participants’ ethnic groups.

| Ethnic Group | N | Percentage of sample |
| --- | --- | --- |
| Arab | 1 | 0.33% |
| Asian Australian | 1 | 0.33% |
| Asian Bangladeshi | 1 | 0.33% |
| Asian Filipino | 1 | 0.33% |
| Asian Indian | 4 | 1.31% |
| Black African | 30 | 9.80% |
| Black American | 1 | 0.33% |
| Black Caribbean | 1 | 0.33% |
| Black Caribbean, White, and Caribbean Indigenous | 1 | 0.33% |
| Chinese | 2 | 0.65% |
| Dutch and New Zealand European | 1 | 0.33% |
| Hispanic/Latinx | 4 | 1.31% |
| Indigenous Mexican | 1 | 0.33% |
| Japanese | 35 | 11.44% |
| Lebanese | 1 | 0.33% |
| New Zealand European (Pākehā) | 16 | 5.23% |
| New Zealand European (Pākehā), and Māori | 3 | 0.98% |
| South African | 1 | 0.33% |
| White | 7 | 2.29% |
| White American | 4 | 1.31% |
| White and Asian | 4 | 1.31% |
| White and Black African | 4 | 1.31% |
| White and Black Caribbean | 2 | 0.65% |
| White and Indigenous North American | 1 | 0.33% |
| White and Native American | 1 | 0.33% |
| White Ashkenazi Jewish | 1 | 0.33% |
| White Australian | 1 | 0.33% |
| White Australian | 6 | 1.96% |
| White Canadian | 1 | 0.33% |
| White Dutch | 2 | 0.65% |
| White English / Welsh / Scottish / Northern Irish / British | 91 | 29.74% |
| White English, Irish, Scottish and Dutch | 1 | 0.33% |
| White European | 3 | 0.98% |
| White European - Greek, German, and Polish | 1 | 0.33% |
| White German | 1 | 0.33% |
| White German | 1 | 0.33% |
| White German and French | 1 | 0.33% |
| White German and Irish | 1 | 0.33% |
| White Greek | 1 | 0.33% |
| White Irish | 5 | 1.63% |
| White Irish and Native Canadian | 1 | 0.33% |
| White Italian | 2 | 0.65% |
| White Mixed | 3 | 0.98% |
| White Polish | 2 | 0.65% |
| White Sardinian, Italian and Ashkenazi | 1 | 0.33% |
| White Scottish, German and Australian | 1 | 0.33% |
| White Slavic | 1 | 0.33% |
| White South African | 5 | 1.63% |
| White Spanish, Iberian, Southern European, and Mediterranean | 1 | 0.33% |
| White Ukrainian | 1 | 0.33% |
| White Ukrainian, Russian and Polish | 1 | 0.33% |
| White Western European | 2 | 0.65% |
| Not disclosed | 40 | 13.07% |
