# Supplementary material for: Cross-cultural variation in experiences of acceptance, camouflaging and mental health difficulties in autism: A registered report
Source: PLoS One. 2024 Mar 20;19(3):e0299824. doi: 10.1371/journal.pone.0299824 (PMC10954134; doi:10.1371/journal.pone.0299824)
Supplement: S2 File — Item and factor analyses on the translated autism acceptance questions and CAT-Q. (DOCX) [file pone.0299824.s002.docx]

**Supporting Information**

**S2 File – Item and factor analyses on translated versions of the scales**

For all item analyses, we used McDonalds omega coefficient as suggested by Revelle and Zinbarg [1] and Beland et al. [2]. The French version of the CAT-Q had good internal consistency for the total scale [McDonalds ωt =0.93] and across all three subscales; compensation [McDonalds ωt = 0.85] masking [McDonalds ωt = 0.86], and assimilation [McDonalds ωt = 0.83]. Similarly, the French [McDonalds ωt = 0.66] and Japanese [McDonalds ωt = 0.77] versions of the autism acceptance questions had acceptable internal consistency, comparable to the scores in Cage et al. [3].

To assess the factor structure of the French version of the CAT-Q, a Principal Component Analysis restricted to three factors was conducted on all 25 items (as in [4]). This revealed that 59.7% of the total variance was explained by the factors. The Eigenvalues for each factor was 9.43, 3.20, and 2.29, respectively. Factor one included five masking items, three compensation items, and two assimilation items; factor two included five assimilation items, three masking items, and one compensation item; and factor three included four compensation items, and one assimilation item. Therefore, although there were some parallels in structure, the French version of the CAT-Q did not display the same factor structure as the English version of the CAT-Q in Hull et al. [5].

To assess the factor structure of the French and Japanese versions of the autism acceptance questions, Principal Component Analyses were conducted on three items. In line with Cage et al. [3], the analysis with the Japanese version extracted one component (Eigenvalue = 1.82), explaining 60.7% of the total variance. External acceptance from society (factor loading = 0.83) and external acceptance from family and friends (factor loading = 0.90) loaded strongly onto this component, while personal acceptance loaded more weakly onto it (factor loading = 0.57). Similarly, the analysis with the French version extracted one component (Eigenvalue = 1.76), explaining 58.6% of the total variance. For this version, external acceptance from society loaded strongly onto the component (factor loading = 0.85), along with external acceptance from family and friends (factor loading = 0.72) and personal acceptance (factor loading = 0.71). These results are comparable to those found in Cage et al. [3].

In sum, these results suggest that the translated versions of the CATQ and autism acceptance questions have good reliability but differing factor structures to the English versions. Therefore, although the total scores on these scales appear to be reliable indicators of acceptance and camouflaging, caution should be advised when interpreting the individual subscale scores. As a result, in the current study we did not conduct any analyses involving the individual subscales of the CAT-Q or individual items relating to external acceptance.

**References**

1. Revelle W, Zinbarg RE. Coefficients alpha, beta, omega, and the glb: Comments on Sijtsma. Psychometrika. 2009 Mar 1;74(1):145-54.
2. Béland S, Cousineau D, Loye N. Utiliser le coefficient omega de McDonald à la place de l’alpha de Cronbach. McGill Journal of Education. 2017;52(3):791-804.
3. Cage E, Di Monaco J, Newell V. Experiences of Autism Acceptance and Mental Health in Autistic Adults. Journal of Autism and Developmental Disorders. 2018;48(2):473–84. Available from: http://dx.doi.org/10.1007/s10803-017-3342-7
4. Hongo M, Oshima F, Guan S, Takahashi T, Nitta Y, Seto M, Hull L, Mandy W, Ohtani T, Tamura M, Shimizu E. Reliability and Validity of the Japanese Version of the Camouflaging Autistic Traits Questionnaire. Pre-print.
5. Hull L, Mandy W, Lai MC, Baron-Cohen S, Allison C, Smith P, et al. Development and Validation of the Camouflaging Autistic Traits Questionnaire (CAT-Q). Journal of Autism and Developmental Disorders. 2019;49(3):819–33. Available from: http://dx.doi.org/10.1007/s10803-018-3792-6
