# Supplementary material for: Cross-cultural variation in experiences of acceptance, camouflaging and mental health difficulties in autism: A registered report
Source: PLoS One. 2024 Mar 20;19(3):e0299824. doi: 10.1371/journal.pone.0299824 (PMC10954134; doi:10.1371/journal.pone.0299824)
Supplement: S3 File — Exploratory regression analyses assessing the relationships between our variables of interest. (DOCX) [file pone.0299824.s003.docx]

**Supporting Information**

**S3 File - Exploratory regression analyses**

*Exploratory analysis: Is acceptance from society or acceptance from family and friends related to depression?*

In order to identify whether a specific type of external acceptance is associated with depression, we conducted an additional exploratory analysis in which we modelled depression scores as a function of acceptance from friends and family, societal acceptance, personal acceptance (independent variables) age, age of diagnosis, gender, stress, anxiety (control variables). This analysis identified that acceptance from family and friends [t(292) = -2.55, p = .011], personal acceptance [t(292) = -3.10, p = .002], stress [t(292) =7.24, p < .0001] and anxiety [t(292) = 4.17, p < .0001] were associated with depression.

*Exploratory analysis: Is camouflaging associated with depression?*

In Hull et al. [1], there was a small contribution of camouflaging to depression scores (*b* = 0.12), however, we did not include this as a formal analysis in our registered report due to concerns over having enough statistical power to detect this effect at p < .0125. Nevertheless, we conducted another hierarchical regression analysis, this time including depression as the outcome variable (as in Hull et al. [1]). The variables in step one (age and AQ) could account for 1.46% of the variance, however, neither was significantly associated with depression [p > .0125]. In step two, adding camouflaging significantly improved the model [F(1) = 15.17, p = .0001, R^2^ change = 4.10%], accounting for an addition 4.40% of the variance in depression scores; as in Hull et al. [1], those who camouflaged their autistic traits to a larger degree exhibited greater depression.

*Exploratory analysis: Is camouflaging associated with stress?*

Studies have not yet assessed whether camouflaging one’s autistic traits is associated with higher levels of stress. As such, we conducted one final hierarchical regression (following the procedures of Hull et al. [1] for depression and anxiety) to test this possibility. The variables in step one (age and AQ) could account for 6.52% of the variance in stress; whilst the level of autistic traits was significantly related to stress [t(303) = 4.76, p < .0001], age was not [p > .0125]. In step two, adding camouflaging significantly improved the model [F(1) = 31.03, p < .0001, R^2^ change = 8.43%], accounting for an additional 8.43% of the variance in stress scores; those who camouflaged their autistic traits to a greater extent displayed greater stress.

*Exploratory Analysis: Do the strengths of associations between our variables of interest differ across our country groups or genders?*

Finally, in order to compare the strengths of association between our variables of interest (external acceptance, personal acceptance, camouflaging, depression, anxiety, and stress) across our country groups, we constructed a series of simple linear models. In instances where the data did not meet parametric assumptions, we conducted these linear models on ranked data (in analyses with depression and anxiety as the outcome variables). In the first three models, depression, anxiety, and stress were the outcome variables, respectively, and the predictor variables were country, external acceptance, personal acceptance, camouflaging, the external acceptance x country interaction, the personal acceptance x country interaction, and finally the camouflaging x country interaction. In the fourth model, camouflaging was the outcome variable and country, external acceptance, personal acceptance, the external acceptance x country interaction, and the personal acceptance x country interaction were predictors. In these analyses, a significant interaction suggests that the strength of the contribution of the predictor variable to the outcome variable differs across countries.

In the first model, depression was considered as the outcome variable. This analysis identified a significant country x personal acceptance interaction [F(7,274) = 3.32, p = .0021], but no other interactions [ps > .05]. These results suggest that personal acceptance may be more strongly related to depression in some countries than others. Unpacking this interaction revealed that personal acceptance was more strongly (negatively) associated with depression in the UK than in Australia [t(274) = 3.61, p = .0087], Belgium [t(274) = 3.28, p = .0260], Japan [t(274) = 3.93, p = .0027], and South Africa [t(274) = 3.21, p = .0319; see Figure 4]. Whilst personal acceptance was associated with depression in the UK [F(1,38) = 37.59 p < .0001], it was not in Australia [p =.651], Belgium [p = .612], Japan [p = .539], and South Africa [p = .363].

**

***Figure 4.*** The contribution of personal acceptance to depression across each country group.

In our models predicting anxiety, stress, and camouflaging, there were no significant interactions with country [all p > .05], thus suggesting that the associations discussed previously (e.g., camouflaging being associated with depression, anxiety, and stress, in respective models) were identified across all country groups (and to a similar extent).

Next in order to assess the strengths of the relationships between our variables of interest across genders, we ran the same analyses again, this time entering the interactions with gender (rather than country group). In all of the analyses, which included depression, anxiety, stress, and camouflaging as the outcome variables, there were no significant interactions with gender. These results suggest that the associations found previously (e.g., personal acceptance associated with depression, camouflaging associated with depression, anxiety, and stress, respectively) were consistent (and comparable in strength) across genders.

*Exploratory Analysis: Does camouflaging mediate the relationship between autism acceptance and mental health.*

Previous research has suggested that camouflaging may mediate the relationship between autism acceptance and mental health (e.g., depression; Cage et al. [2]). Therefore, we conducted two mediation analyses to test this. There are several methods used to test the statistical significance of mediated effects [3-5]. In the present study, we used structural equation modelling (SEM), a powerful statistical technique used to infer causal relationships between variables, for our mediation analyses. We decided to use SEM over standard regression methods (as outlined by Baron and Kenny; [6]) as this technique is regarded as “a more appropriate inference framework for mediation analyses” (see [7]). In addition, we used bias-corrected bootstrapping to create confidence intervals as there is a consensus that it is the most powerful method for testing mediated effects [3, 8-11].

In the first model, we modelled external acceptance as the predictor, camouflaging as the mediator and depression as the outcome variable. In the second, we replaced external acceptance with personal acceptance. In the first model, although there were direct effects of external acceptance on depression [z = -5.14, p < .001, *b =* -0.29, 95% Confidence Intervals(CI) = (-2.00, -0.90)] and camouflaging on depression [z = 3.98, p < .001, *b =* 0.23, 95% CI = (0.06, 0.17)], there was not a significant direct effect of external acceptance on camouflaging [z = -1.45, p = .148, *b* = -0.08, 95% CI = (-1.96, 0.30)], nor a significant indirect effect [z = -1.36, p = .175, *b* = -0.02, 95% CI = (-0.25, 0.03)]. Similarly, in the second model, whilst there was a direct effect of personal acceptance on depression [z = -5.75, p < .001, *b* = -0.31, 95% CI = (-1.86, -0.91)], and a direct effect of camouflaging on depression [z = 4.35, p < .001, *b* = 0.25, 95% CI = (0.07, 0.18)], there was not a significant direct effect of personal acceptance on camouflaging [z = -0.10, p = .920, *b* = -0.01, 95% CI = (-1.19, 1.05)]. In addition, there was not a significant indirect effect of personal acceptance on depression [z = -0.10, p = .921, *b* = 0.00, 95% CI = (-0.15, 0.14)]. Therefore, camouflaging did not mediate the relationship between external acceptance and depression, or personal acceptance and depression.

**References**

1. Hull L, Levy L, Lai MC, Petrides K V., Baron-Cohen S, Allison C, et al. Is social camouflaging associated with anxiety and depression in autistic adults? Molecular Autism. 2021;12(1):13. Available from: https://molecularautism.biomedcentral.com/articles/10.1186/s13229-021-00421-1
2. Cage E, Di Monaco J, Newell V. Experiences of Autism Acceptance and Mental Health in Autistic Adults. Journal of Autism and Developmental Disorders. 2018;48(2):473–84. Available from: http://dx.doi.org/10.1007/s10803-017-3342-7
3. Hayes AF, Scharkow M. The Relative Trustworthiness of Inferential Tests of the Indirect Effect in Statistical Mediation Analysis: Does Method Really Matter? Psychological Science. 2013; 24(10):1918–27. Available from: http://journals.sagepub.com/doi/10.1177/0956797613480187
4. MacKinnon DP, Lockwood CM, Hoffman JM, West SG, Sheets V. A comparison of methods to test mediation and other intervening variable effects. Psychological Methods. 2002;7(1):83–104. Available from: /record/2002-00925-005
5. Shrout PE, Bolger N. Mediation in experimental and nonexperimental studies: New procedures and recommendations. Psychological Methods. 2002;7(4):422–45. Available from: /record/2002-11349-006
6. Baron RM, Kenny DA. The moderator-mediator variable distinction in social psychological research: Conceptual, strategic, and statistical considerations. Journal of Personality and Social Psychology. 1986;51(6):1173–82. Available from: https://pubmed.ncbi.nlm.nih.gov/3806354/
7. Gunzler D, Chen T, Wu P, Zhang H. Introduction to mediation analysis with structural equation modeling. Shanghai Archives of Psychiatry. 2013;25(6):390–4. Available from: /pmc/articles/PMC4054581/
8. Cheung MWL. Comparison of Approaches to Constructing Confidence Intervals for Mediating Effects Using Structural Equation Models. Structural Equation Modeling: A Multidisciplinary Journal. 2007;14(2):227–46. Available from: http://www.tandfonline.com/doi/abs/10.1080/10705510709336745
9. Fritz MS, MacKinnon DP. Required sample size to detect the mediated effect. Psychological Science. 2007;18(3):233–9. Available from: http://journals.sagepub.com/doi/10.1111/j.1467-9280.2007.01882.x
10. MacKinnon DP, Lockwood CM, Williams J. Confidence limits for the indirect effect: Distribution of the product and resampling methods. Multivariate Behavioral Research. 2004;39(1):99–128. Available from: https://www.tandfonline.com/doi/abs/10.1207/s15327906mbr3901_4
11. Valente MJ, Gonzalez O, Miočević M, MacKinnon DP. A Note on Testing Mediated Effects in Structural Equation Models: Reconciling Past and Current Research on the Performance of the Test of Joint Significance. Educational and Psychological Measurement. 2016;76(6):889–911. Available from: http://www.ncbi.nlm.nih.gov/pubmed/27833175
