# Supplementary material for: Cross-cultural variation in experiences of acceptance, camouflaging and mental health difficulties in autism: A registered report
Source: PLoS One. 2024 Mar 20;19(3):e0299824. doi: 10.1371/journal.pone.0299824 (PMC10954134; doi:10.1371/journal.pone.0299824)
Supplement: S4 File — The association between age, age of diagnosis, autistic traits, alexithymic traits, gender, sexuality, level of education, current verbal level, and the presence of co-occurring conditions with our variables of interest. (DOCX) [file pone.0299824.s004.docx]

**Supporting Information**

**S4 File – Determining the relationships between our variables of interest and control variables.**

***Continuous individual differences***

In order to assess the contribution of various individual differences to acceptance, camouflaging and mental health, we conducted a) spearman’s correlations between our outcome variables and continuous individual difference variables (i.e., age, age of diagnosis, autistic traits, alexithymic traits; see Table S1.), and b) Multivariate Kruskal-Wallis tests assessing the contribution of our categorical individual difference variables (e.g., gender, sexuality, level of education, income, etc.) to our outcome variables.

This revealed that there were significant correlations between age and external acceptance [r = -.196, p < .001], age of diagnosis and external acceptance [r = -.292, p < .001], autistic traits and external acceptance [r = -.222, p < .001], alexithymic traits and external acceptance [r = -.149, p = .009], age of diagnosis and camouflaging [r = .125, p = .028], autistic traits and camouflaging [r = .347, p < .001], alexithymic traits and camouflaging [r = .310, p < .001], autistic traits and stress [r = .234, p < .001], and alexithymic traits and depression [r = .257, p < .001], anxiety [r = .335, p < .001], and stress [r = .464, p < .001] respectively. Therefore, we controlled for age, age of diagnosis, autistic traits and alexithymic traits in our subsequent ANCOVA analyses comparing levels of external and personal acceptance, camouflaging, depression, anxiety, and stress across our country groups.

***Table S1.*** The relationships between our primary variables of interest and continuous individual difference variables.

|  | External acceptance | Personal acceptance | Camouflaging | Depression | Anxiety | Stress |
| --- | --- | --- | --- | --- | --- | --- |
| Age | r = -.196  p < .001*** | r = .088  p = .127 | r = -.029  p = .613 | r = -.044  p = .448 | r = -.099  p = .085 | r = -.021  P = .714 |
| Age of diagnosis | r = -.292  p < .001*** | r = .027  p = .641 | r = .125  p = .028* | r = -.021  p = .720 | r = -.095  p = .099 | r = .050  p = .381 |
| Autistic traits | r = -.222  p < .001*** | r = .096  p = .094 | r = .381  p < .001*** | r = .069  p = .228 | r = .080  p = .161 | r = .234  p < .001*** |
| Alexithymic traits | r = -.149  p = .009** | r = -.057  p = .320 | r = .324  p < .001*** | r = .257  p < .001*** | r = .335  p < .001*** | r = .464  p < .001*** |

***Categorical individual differences***

For the following analyses, in which we assess whether our outcome variables differ across groups of categorical demographic variables, we only include groups that contain at least 20 participants (and thus that have enough data to conduct statistical comparisons).

*Gender*

Our first multivariate Kruskal Wallis test revealed a significant effect of gender on our outcome variables [H(12) = 62.48, p < .0001]. Following this, we unpacked this effect using univariate Kruskal-Wallis tests which revealed a significant effect of gender for external acceptance [H(2) = 15.80, p < .001], camouflaging [H(2) = 35.28, p < .0001], and stress [H(2) = 15.78, p <.001], but no other variables. Individuals identifying as males experienced the highest levels of external acceptance [mean(SEM) = 5.89(0.22)] and the lowest levels of camouflaging [109.9(2.16)] and stress [mean(SEM) = 19.34(0.92)], whilst those identifying as non-binary or a third gender experienced the lowest levels of acceptance [mean(SEM) = 4.21(0.40)] and the highest levels of camouflaging [mean(SEM) = 132.8(3.39)] and stress [mean(SEM) = 27.69(1.88)] (with those identifying as female as an intermediate level [external acceptance mean(SEM) = = 5.23(0.18); camouflaging mean(SEM) = 123.8(1.95) ; stress mean(SEM) = 22.95(0.95)]).

*Sexuality*

Our multivariate Kruskal Wallis test also identified a significant effect of sexuality on our outcome variables [H(12) = 34.13, p < .001]. Unpacking this effect with univariate Kruskal Wallis tests revealed a significant effect of sexuality specifically on camouflaging [H(2) = 16.17, p < .001] and stress [H(2) = 6.39, p = .041], but no other variables. Bonferroni-corrected Dunn’s tests revealed that heterosexual individuals camouflaged their autistic traits [mean(SEM) = 112.7(1.88)] least, followed by gay or lesbian [mean(SEM) = 123.3(3.98)] and bisexual [mean(SEM) = 127.1(3.05)] individuals. In addition, heterosexual [mean(SEM) = 20.98(0.86)] and bisexual [mean(SEM) = 22.51(1.21)] individuals experienced the lowest levels of stress followed by gay or lesbian people [mean(SEM) = 26.14(1.90)].

*Level of education*

In addition, our multivariate Kruskal Wallis test revealed a significant effect of level of education on our outcome variables [H(24) = 44.90, p = .006]. Unpacking this effect revealed a significant effect of level of education on camouflaging [H(4) = 11.86, p = .018] and depression [H(4) = 10.87, p = .028], but no other variables. Individuals whose highest level of education is upper secondary education camouflaged their autistic traits the most [mean(SEM) = 108.42(2.99)], followed by Master’s or equivalent level [mean(SEM) = 117.6(3.23)], Bachelor’s or equivalent level [mean(SEM) = 121.3(2.31)], lower secondary education [mean(SEM) = 122.5(3.32)] and post-secondary non-tertiary education [mean(SEM) = 122.5(4.32)]. Moreover, those whose highest level of education is lower secondary education reported the highest levels of depression [mean(SEM) = 24.64(1.48)], followed by Bachelor’s or equivalent level [mean(SEM) = 19.71(1.16)], upper secondary education [mean(SEM) = 19.55(1.44)], post-secondary non-tertiary education [mean(SEM) = 18.73(2.33)], and Master’s or equivalent level [mean(SEM) = 17.73(1.83)].

*Current verbal level*

Furthermore, our multivariate Kruskal Wallis tests revealed a significant effect of current verbal level on our outcome variables [H(6) = 25.88, p = .0002]. Univariate Kruskal Wallis tests identified that current verbal level had a significant effect on anxiety [H(1) = 21.31, p < .0001] and stress [H(1) = 4.94, p = .026]. Minimally verbal individuals displayed significantly higher anxiety [mean(SEM) = 25.53(1.68)] and stress [mean(SEM) = 26.32(1.63)], than verbal individuals [anxiety mean(SEM) = 15.77(0.65); stress mean(SEM) = 21.15(0.68)].

*Co-occurring conditions*

In addition, our multivariate Kruskal Wallis tests revealed a significant effect of having co-occurring conditions on our outcome variables [H(6) = 23.92, p = .0005]. Unpacking this with univariate Kruskal Wallis tests identified that having co-occurring conditions had a significant effect on external acceptance [H(1) = 12.99, p = .0003], camouflaging [H(1) = 7.16, p = .0074], depression [H(1) = 8.55, p = .0035], and stress [H(1) = 7.67. p = .0056]. Those with co-occurring conditions experienced significantly lower external acceptance [mean(SEM) = 5.01(0.18)], and higher camouflaging [mean(SEM) = 122.0(1.75)], depression [21.62(0.89)], and stress [mean(SEM) = 23.26(0.80)], than those without co-occurring conditions [external acceptance mean(SEM) = 6.00(0.21); camouflaging mean(SEM) = 112.9(2.23); depression mean(SEM) = 17.41(1.09); stress mean(SEM) =19.73(1.02)].

*Income, number of siblings,*

Finally, our multivariate Kruskal Wallis tests revealed no significant effect of income or number of siblings on our outcome variables [both p > .05].

In light of these significant relationships and effects, we controlled for age, age of diagnosis, autistic traits, alexithymic traits, gender, sexuality, level of education, verbal level, and presence of co-occurring conditions in the following analyses comparing our outcome variables across country groups (see main manuscript).
