# Supplementary material for: Cross-cultural variation in experiences of acceptance, camouflaging and mental health difficulties in autism: A registered report
Source: PLoS One. 2024 Mar 20;19(3):e0299824. doi: 10.1371/journal.pone.0299824 (PMC10954134; doi:10.1371/journal.pone.0299824)
Supplement: S5 File — (DOCX) [file pone.0299824.s005.docx]

**Supporting Information**

**S5 File – Estimated prevalence of autism across countries.**

***Table S1.*** Estimated prevalence of autism across the studied countries [1].

| **Country** | **Estimated Prevalence** |
| --- | --- |
| Australia | 1 in 113 |
| Belgium | 1 in 137 |
| Canada | 1 in 116 |
| Japan | 1 in 117 |
| New Zealand | 1 in 117 |
| South Africa | 1 in 120 |
| United Kingdom | 1 in 128 |
| United States | 1 in 124 |

References

1. World Population Review. Autism Rates by Country 2023 [Internet]. Available from: <https://worldpopulationreview.com/country-rankings/autism-rates-by-country>
